# Feasibility assessment of an ergonomic baby wrap for kangaroo mother care: A mixed methods study from Nepal

**DOI:** 10.1371/journal.pone.0207206

**Published:** 2018-11-15

**Authors:** Kusum Thapa, Diwakar Mohan, Emma Williams, Chandra Rai, Sangita Bista, Sangeeta Mishra, Pawan Kumar Hamal

**Affiliations:** 1 Maternal and Child Survival Program/Jhpiego, Washington, DC, United States of America; 2 Johns Hopkins Bloomberg School of Public Health, Baltimore, Maryland, United States of America; 3 Jhpiego/Nepal, Oasis Building, Patan Dhoka, Lalitpur, Kathmandu, Nepal; 4 Koshi Zonal Hospital, Biratnagar, Nepal; 5 National Academy of Medical Sciences, National Trauma Center, Kathmandu, Nepal; ESIC Medical College & PGIMSR, INDIA

## Abstract

**Background:**

Kangaroo mother care, an evidence based practice and a national policy for management of low birth weight newborns in Nepal, is not widely practiced. This implementation research study aimed to explore the consumer preference and acceptability of the traditional and a new ergonomic wrap on the continuation of kangaroo mother care in the facility and community following discharge.

**Methods:**

A mixed method feasibility study was done from May to October 2015. Ninety-six families of stable low birth weight newborns weighing 1800 to 2499 grams were counseled and taught to practice kangaroo mother care using both wraps. They were randomized into two groups of 48 with one group trying out the traditional wrap for the first six hours and the new wrap for the next six, and vice versa. Mothers were allowed to choose between the wraps for continuation of kangaroo mother care at the facility and post discharge. They were followed up telephonically weekly over 28 days postpartum to ascertain practice of kangaroo mother care. In-depth interviews with mothers (n = 12) and focus group discussions with health workers (n = 16) further evaluated the intervention. Descriptive statistics are presented for the quantitative part of the study.

**Results:**

Mothers in the two groups chose the new wrap with no significant difference (81.3% vs 89.6%, p = 0.24). Of the 96 randomized mothers, 85% chose the new wrap. During the hospital stay, six mothers dropped out and remaining 90 mothers who were discharged with the intention of continuing Kangaroo Mother Care, 78 and 12 mothers did so with the new and traditional wrap respectively. New wrap users (429.1 hours, 95% confidence interval [CI]: 351.7–470.3) performed skin-to-skin contact for an extra 77.4 hours overall than traditional wrap (351.7 hours, 95%CI: 259.3–444) users from first day to 28 day postpartum. Health workers and mothers reported positive experience with the new wrap as it was easy to wear without assistance, secure and flexible to move around in kangaroo mother care position.

**Conclusions:**

Involvement of mothers and families with provision of ergonomic wraps showed improvement in kangaroo mother care practice during hospital stay and at home.

## Introduction

The neonatal mortality rate at the global level fell from 36 per 1,000 live births in 1990 to 19 in 2015, but this decrease in neonatal mortality has been slower than that of post-neonatal under-five mortality: 47%, vs 58% [1]. The Sustainable Development Goal (SDG) aims to reduce neonatal mortality to as low as 12 per 1000 live birth [2]. Low birth weight (LBW),due to preterm birth and/or small for gestational age (SGA), accounts for more than 80% of neonatal deaths worldwide as well as increasing the risk of post-neonatal mortality [3]. In Nepal, newborn mortality, over the past 15 years, has declined at a slower pace than infant and child mortality overall, resulting in an increase in the proportion of neonatal mortality of all infant deaths (63% in 1996 to 72% in 2011) and under-five deaths (42% to 61%). According to the Nepal Demographic Health Survey (NDHS) 2011, approximately 12% of infants with reported weight were classified as LBW [4]. Nepal must reduce neonatal mortality to less than 11 per 1,000 live births in every province by 2035 to achieve the goal of Nepal’s Every Newborn Action Plan (NENAP) [5].

Kangaroo Mother Care (KMC)—defined as early, prolonged skin-to-skin (STS) contact between mothers, family members and the newborn with exclusive or near exclusive breastfeeding along with early discharge from hospital has been proposed as an alternative to incubator care for LBW infants [6]. KMC has been shown to be the most feasible, readily available, and preferred intervention for decreasing newborn morbidity and mortality in developing countries; especially for LBW newborns [7–8].The NENAP has set a target to increase the proportion of all premature newborns receiving KMC to 35% by 2035 from the present situation of 0% coverage [5].

KMC has been applied in different ways in different contexts, and studies show that there are several barriers to implement KMC, including the need for time, social support, medical care and family acceptance [9–10]. Success of implementation requires high user engagement and stakeholder’s involvement, as KMC is a complex behavior-driven intervention and includes multiple elements [9]. A systematic review noted that family members play a vital role in KMC adoption, as they are the primary caregivers for LBW newborns and involved in decision making for practice of care [9]. Their conviction that the intervention is good for the newborn improved their motivation to practice KMC and promoted its adoption, while limited understanding about KMC, primarily due to lack of counseling or limited health workers competency in technical and counselling, hindered its practice [9–10]. Additional barriers to KMC included “pain and fatigue” among mothers due to the weight of the newborn, positioning issues while sleeping with the infant, discomfort during warm weather, and issues with mother’s clothing [11]. In our review of the literature on KMC, we did not find any studies about the influence of the physical characteristics of the wrap to hold the newborn positioned on a caregiver’s chest on KMC acceptance.

Despite strong evidence about improved health outcomes among LBW newborns receiving KMC, it has never been completely integrated within the health systems globally and this holds true for Nepal [5, 10]. Though KMC is a national policy for care of LBW newborns in Nepal and being included in preservice and in-service curriculum for health workers, it is not widely practiced or promoted. Nepal Ministry of Health, in collaboration with Jhpiego and Laerdal Global Health, conducted an implementation research study in two peripheral hospitals of Nepal. The primary objective of the study was to understand the consumer preference and acceptability of the traditional and a new ergonomic wrap on the continuation of KMC in the facility and community following discharge.

## Materials and methods

### Study setting

We implemented the study in two peripheral hospitals of Morang and Jhapa districts in the Eastern Development Region of Nepal from May to October 2015. We selected this region due to high prevalence of LBW (16%), compared to the national average (12%) [4]. The hospitals were selected because of their high annual volume of births, which were 9,000 and 6,000 per year, respectively. The health workers of these two hospitals received two days training on KMC using the national training package for the care of LBW newborns.

### Study design

The study was designed as a sequential explanatory design, with a trial for mothers of LBW newborns adopting KMC practice using different wraps, followed by collection and analysis of qualitative data on perceptions and experiences among providers and mothers. The reason for the above design was to use qualitative results to assist in explaining and interpreting the findings of the initial trial [12]. The need for implementation research is to understand not only what works, but also the reasons for how implementation is proceeding the way it is, and testing the approaches to improve it [13]. The study is structured using the Consolidated Framework for Implementation Research (CFIR), which has been used to analyze the characteristics of promising health interventions [14]. Although CFIR comprises five major domains -*intervention*, *inner and outer settings*, *individuals involved*, *and the process by which implementation is accomplished* -we focus our efforts on the stakeholder perceptions, adoption and trialability of the intervention.

### Intervention

**Fig 1:**
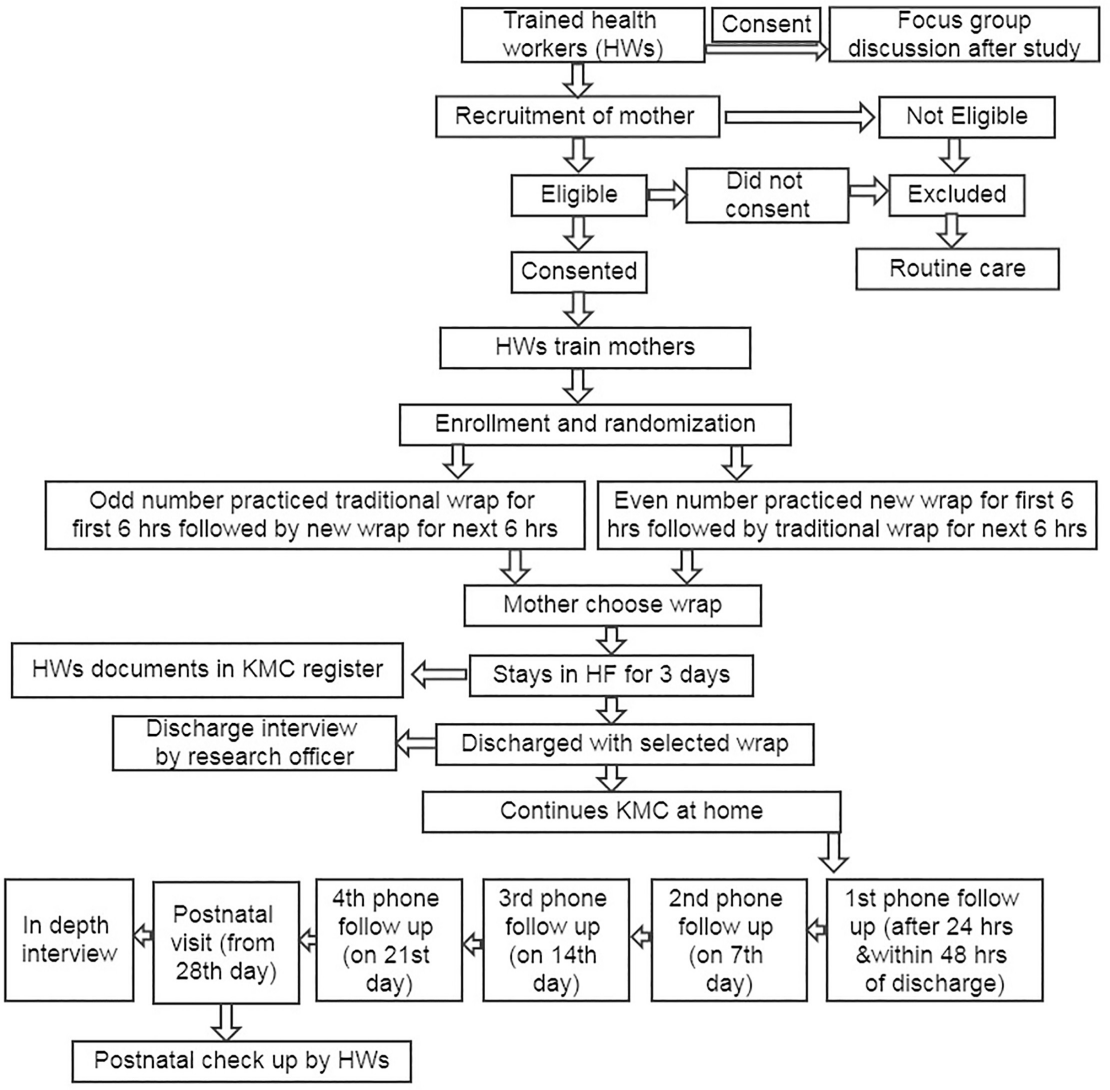
Intervention design of the study.

The intervention is pictorially represented (Fig 1). Health workers identified mothers with LBW newborns in the selected health facilities and informed them about the weight of the newborn and importance of KMC. They screened mothers with stable, pre-term (<37 weeks gestational age) and/or LBW newborns (>1800 and < 2500 grams). The enrollment was restricted to subjects who provided informed consent to participate and agreed to be interviewed by phone weekly over the four-week postnatal period and return for a postnatal visit. We excluded mothers who experienced any complications during childbirth that resulted in obvious pain or who were sedated, delivered by cesarean section or had a newborn sick enough to be admitted to the newborn care unit. Mothers who did not meet eligibility criteria or did not provide consent received routine conventional care.

We randomized mothers based on odd and even numbers in the sequence of enrollment. A coin was flipped to determine the group allotment for the first enrollee in both the facilities, which ended up as the traditional wrap first. Subsequently, all the odd numbers received traditional wrap first and even numbers received the new wrap first in both facilities. After obtaining informed consent from the mother, the health worker counseled the mother and other family members (if present) on the benefits of KMC and demonstrated the steps of correct KMC using both the wraps. Mothers and family members of the newborn practiced KMC with Preemie Natalie (Laerdal preterm simulation model) using both the wraps. After the mother and other family members were comfortable and confident in performing KMC, they practiced KMC with both wraps, switching after six hours for a total of 12 hours with the practice on the first one based on the sequence of randomization. They were offered the opportunity to choose one of the wraps to continue practicing KMC at the facility and at home after discharge. The preferred wrap was provided free of cost. All mothers with LBW newborns were encouraged to stay in the health facility for three days to allow for early detection and appropriate management of early newborn sepsis, which usually manifests by the third day.

The two types of wraps used as part of the study are briefly described here.

#### Traditional wrap

The traditional cloth (S1 Fig) for carrying babies on the mother’s chest in Nepal is a three meter long thick and warm flannel cloth. It requires at least one person other than the wearer to help tie and untie while practicing KMC. The traditional wrap is currently used in Nepal for practicing KMC.

#### New ergonomic wrap

The new wrap designed by Laerdal Global Health, also known as CarePlus, is an ergonomic wrap (S2 Fig). The wearer can tie the wrap himself or herself without any extra help. This wrap has a shoulder and a waistband that can be tied by crossing them over at the backs [15]. In this document, we continue to reference this new ergonomic wrap as new wrap.

### Sample size considerations

Since the target population for this study is mothers with LBW newborns requiring KMC, the sample size was based on the feasibility of enrolling LBW newborns within the study period. A *post hoc* power analysis was performed to determine if the choice of wrap by mothers was associated with the sequence, in which the wraps were presented to and tried out by the mothers for six hours. For the randomized experiment, we were interested in testing the hypothesis that the sequence in which the wraps were presented to the women was not associated with the final choice of the type of wrap. A sample of 96 women (48 in each arm) would have rejected the null hypothesis with type 1 error of 0.05 and power of 80% only if the odds of the choice of wrap were at least 56% lower in one group over the other.

We conducted in-depth interview with subset of 12 mothers (6 per facility) who were discharged from the health facility with the intention of continuing KMC, attended four phone follow ups and postnatal care. After the quantitative study was analyzed, we randomly sampled mothers, after stratifying them into high and low KMC practice among users of both wraps. We also conducted one focus group discussion (FGD) at each hospital with 16 health workers who had been involved in the study.

### Data collection

Health workers prospectively recorded the type of wrap chosen and the details of KMC practiced by the mothers in the KMC register during hospital stay. During discharge, the mother was interviewed about the reasons for choosing a particular wrap. The study participants were followed up with phone interviews at 24–48 hours after discharge, 7^th^ day, 14^th^ day and 21^st^ day postnatal at home and an in-person interview during the postnatal visit around the 28^th^ day at hospital. A qualitative researcher completed in-depth interviews with a subset of mothers of LBW newborns and FGDs with health workers involved in the study, to explore their experience with the wraps. During the phone interview, mothers were inquired about duration of STS practiced and their experience with providing KMC. No diary or paper format was used by the mother and family members to document the hours of KMC practiced. We filed and stored the completed tools and informed consent documents in a locked cabinet in the respective hospitals with access restricted to the research officers. The data files was entered and stored in password protected computers, with access limited to research personnel. The copy of the interview guide (S3 and S4 Figs), topic guide (S5 and S6 Figs) and questionnaire (S7 and S8 Figs) used in the study in both the original and English language has been mentioned in supporting information section.

### Data analysis

Categorical data are presented in contingency tables as frequencies and proportions with Pearson chi-squared tests. Non-normal continuous variables are presented as medians with the equality test for medians. Hours of STS are presented as an aggregate sum for the entire duration of 28 days of the neonatal period, averaged per participant. For qualitative data, the interviews and FGDs were recorded and transcribed. A thematic analysis of the qualitative data was completed, and the themes were derived from the study questions.

### Ethical considerations

Ethical approval was obtained from the Johns Hopkins University Institutional Review Board and the Nepal Health Research Council, Kathmandu to conduct the study. All the health workers including the research officers received ethical and data management training. Written informed consent was obtained from mothers who participated in the study and health workers involved in FGD.

## Results

**Fig 2:**
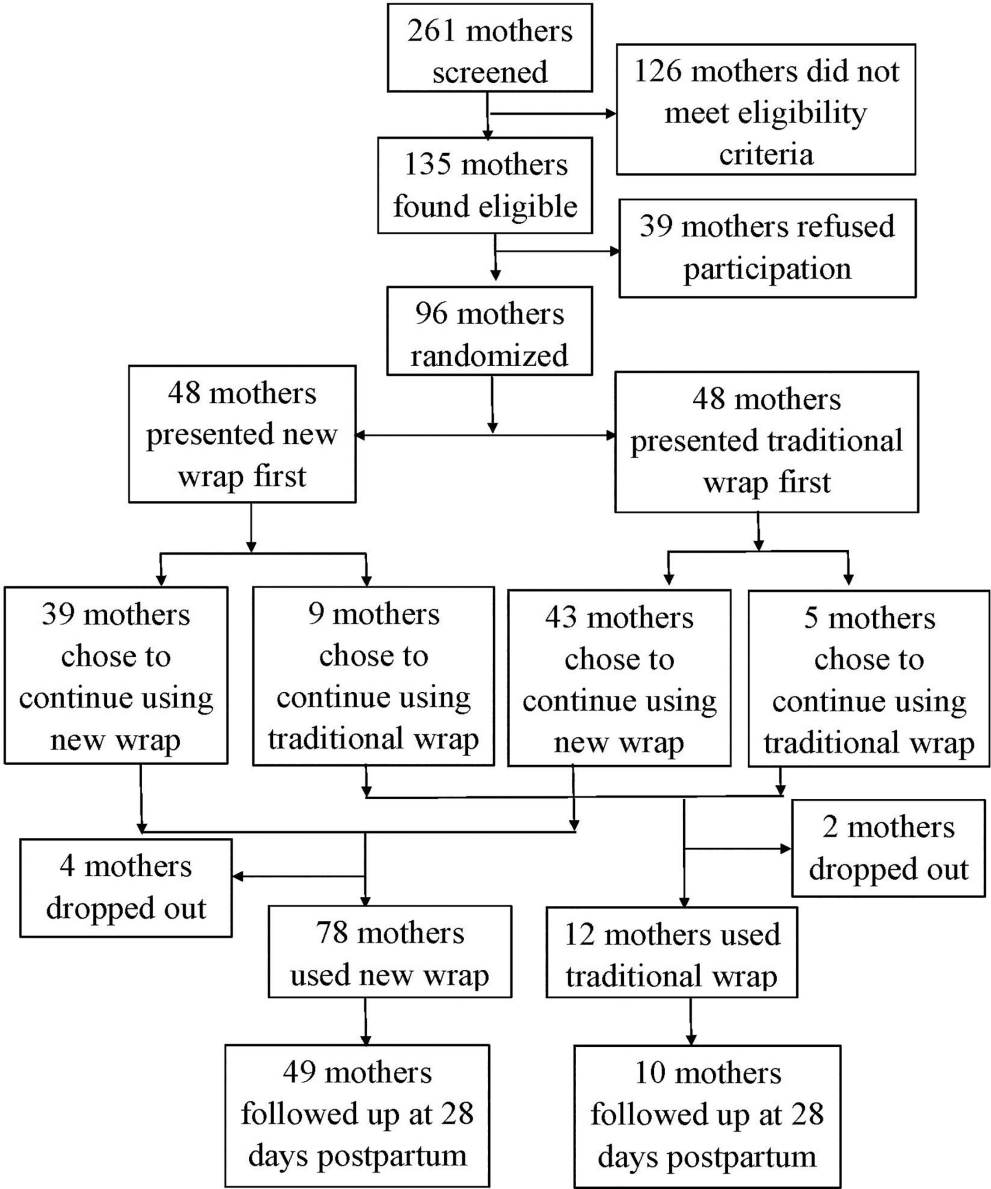
Flow chart of study participants.

Of the 261 mothers screened during the study period, 126 did not meet eligibility criteria, and 39 mothers did not consent for the study while 96 mothers with LBW newborns were enrolled in the study as shown (Fig 2).

Table 1 shows the socio-demographic characteristics of mothers randomized into new wrap and traditional wrap. Mothers participating in the study were predominantly from the same district where the study hospitals were located. They were mainly primipara and were primarily of Dalit, Janjati and Madeshi ethnicity.

**Table 1:** Socio demographic characteristics of mothers randomized in the study (N = 96).

Characteristics	Wraps		Total N	P Value
	Even number (new wrap first) n(%)	Odd number (traditional first) n (%)		
**Residence**				
Study site district	39 (52.7%)	35 (47.3%)	74	0.33
Adjoining district	9 (40.9%)	13 (59.1%)	22	
**Median age(IQR)**	22 (5)	20 (3)		0.08
Caste/Ethnicity*				
Dalit	13(46.4%)	15(53.6%)	28	0.98
Janajati	14(50%)	14(50%)	28	
Madeshi	12(52.2%)	11(47.8%)	23	
Muslim	4(57%)	3(42.9%)	7	
Brhamin/Chhetri	5(50%)	5(50%)	10	
**Parity**				
Primipara	34(48.6%)	36(51.4%)	70	0.81
Multipara	14(53.8%)	12(47.1%)	26	
**Sex of newborn**				
Female	28**(57.1%)	21(42.9%)	49	0.18
Male	21(43.8%)	27(56.3%)	48	
Birth Weight (IQR)	2100(200)	2150(275)	97	0.92
**Selected wrap**				
New Wrap	39(47.6%)	43(52.4%)	82	0.24
Traditional wrap	9(64.3%)	5(35.7%)	14	

*Caste/Ethnicity stratification is a major determinant of people’s identity and social status in Nepalese society.

** one set of twins

Among the 48 mothers who were presented with new wrap first, 39 (81.3%) chose the new wrap and 9 (18.7%) mothers chose the traditional wrap. Similarly, out of 48 mothers who were presented with traditional wrap first, 43 (89.6%) mothers chose the new wrap and 5 (10.4%) mothers chose the traditional wrap. The difference between the proportion choosing the different wraps did not differ significantly between the two groups (p = 0.24). There was no significant differences at baseline between mothers selecting the new wrap and traditional wrap (Table 2).

**Table 2:** Socio-demographic characteristics of enrolled mothers in the study (N = 96).

Characteristics	Wraps		Total N	P Value
	New wrap n (%)	Traditional wrap n (%)		
**Residence**				
Study site district	62(75.6%)	12(85.7%)	74	0.40
Adjoining district	20(24.4%)	2(14.3%)	22	
**Median Age (Range)**	20 (16–37)	21.50 (17–28)		0.70
Caste/Ethnicity*				
Dalit	25(30.5%)	3(21.4%)	28	0.35
Janajati	26(31.7%)	2(14.3%)	28	
Madeshi	18(22.0%)	5(35.7%)	23	
Muslim	6(7.3%)	1(7.1%)	7	
Brhamin/Chhetri	7(8.5%)	3(21.4%)	10	
**Parity**				
Primipara	59(72.0%)	11(78.6%)	70	0.61
Multipara	23 (28.0%)	3 (21.4%)	26	
**Sex of newborn**				
Female	43(52.4%)	6**(40%)	49	0.38
Male	39 (47.6%)	9(60%)	48	
Birth weight (IQR)	2100(300)	2175(250)	97	0.97

*Caste/Ethnicity stratification is a major determinant of people’s identity and social status in Nepalese society.

** one set of twins

Subsequent to the choosing of wraps for continuation of KMC, 4 mothers (5%) from the new wrap group and 2 (14%) from the traditional wrap group dropped out of the study, for various reasons, before being discharged from the hospital. Of the 90 mothers remaining in the study at discharge, one mother was discharged before 3 days, and the remaining 89 mothers were discharged after 3 days. All mothers (90) who were enrolled in the study provided exclusive breastfeeding to their newborns during hospital stay. Overall, 77(86%) mothers were followed up until the 4^th^ phone call, of which 66 continued using the new wrap and 11 with the traditional one, and 59 mothers completed the postnatal contact on 28th day.

### KMC practice with different wraps

**Fig 3:**
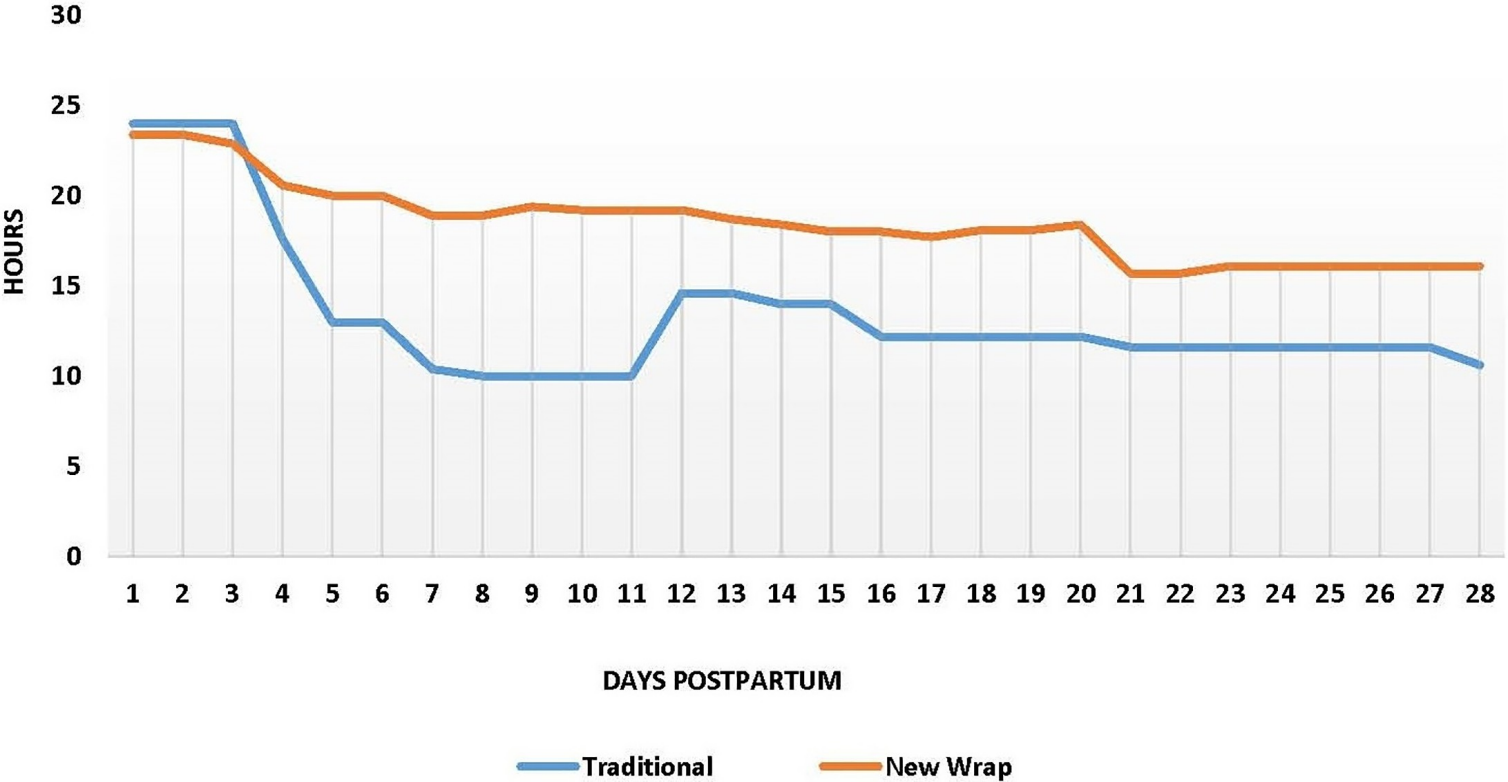
Hours of skin to skin practiced.

The mean duration of STS practiced per day from day 1 to day 28 by type of wrap selected (Fig 3). New wrap users practiced STS care for an average of 429.1 hours (95% confidence interval [CI]: 351.7–470.33) in total, and traditional wrap practiced for 351.7 hours (95% CI: 259.3–443.98) users in total from day 1 postpartum to the 28-day postnatal visit. Therefore, new wrap users reported performing STS contact for an extra 77.4 hours, though we did not achieve statistical significance. The graph (Fig 3) shows, the new wrap and traditional wrap users practiced KMC for nearly about 24 hours when they were in the health facility for three days. The hours of KMC practiced at home has declined gradually over the days in both groups; however, the decline appears steeper in traditional wrap users compared to new wrap users.

Of the 59 (49 new wrap and 10 traditional wrap) mothers who came for postnatal visit, 58 (98%) reported practicing KMC at home. One mother, using traditional wrap, did not practice KMC from day 14 postpartum as the newborn suffered from buttock sores. Among 58 mothers practicing KMC, 55 (94.8%) maintained exclusive breastfeeding until 28 days postpartum, with 2 mothers from new wrap group and 1 from traditional wrap group not breast feeding exclusively.

### Health worker’s perception and practice of KMC

The health workers reported that their perception and understanding about KMC and its benefits before the intervention was limited to STS contact and the treatment of hypothermia. They had not counseled mothers of LBW newborns for KMC, as they were unaware of its benefits. After the intervention, health workers reported the realization that KMC was a standard method of care for LBW newborns which included STS contact and exclusive breastfeeding; perceived benefits included helping maintain the newborn’s body temperature, reducing infection, promoting breastfeeding, and increasing weight gain. They reported counseling mothers of LBW newborns to practice KMC. One of the FGD participants (trained nursing staff) described the change in perception of KMC as,

*“Before the training*, *we just informed the mother to keep the newborn warm*, *but*, *now*, *we counsel them about all aspects of KMC*.*”* Another nursing staff providing KMC added, “*We can answer the queries of visitors about KMC and confidently explain about the benefits”*.

Health workers appeared to have positive attitudes and experiences with the new wrap for KMC in both health facilities. The key characteristics mentioned were that the new wrap was easy to wear without assistance, provided proper support for the newborn because of the shoulder band and, hence, was comfortable to carry the newborn and move around. They also mentioned ease of breastfeeding as the mother can slacken the knot and loosen the strap of one side and feed the newborn herself without help of another people. While in the traditional wrap, there is always the need for another person to release the knot at the back for feeding, thus makes the breastfeeding cumbersome. Health workers also reported that male members found the new wrap acceptable to use on their own. According to their observation at health facility, they found that fathers were more interested and engaged in providing KMC to the newborn with the new wrap as compared to the traditional wrap. They mentioned that the traditional wrap is considered a women’s cloth and men were not willing to use it while the “decent” look of the new wrap promoted its use among male members.

The health workers mentioned that traditional wrap was inconvenient, as another person was always needed to support the mother and help to keep the newborn in KMC. As the traditional wrap was thick and long it was uncomfortable for the mothers during summer due to heat and would sweat considerably. It was not easy to sleep in KMC position with the wrap because of the knot at the back.

### Mother’s perception and practice of KMC

Mothers, irrespective of the wrap they had selected, reported that KMC was beneficial for their LBW newborns and to them as parent. Mothers reported that KMC kept the newborn warm, relaxed, provided more opportunity for breastfeeding and promoted weight gain. Few mothers reported that KMC also reduced the chances of infection in the newborn, as outsiders did not carry them. One mother reported, “*Reduced risk of infection*, *as there is less chance that relatives and neighbors will touch and carry the newborn while the newborn is in KMC*. *If the newborn was not kept in KMC*, *the relatives and neighbors coming to see the newborn would touch and carry the newborn thereby increasing the chances of infection*.*”*

Some mothers expressed being satisfied and happy, as they were able to provide increased care to their newborn. Mothers also said that KMC saved money indirectly as KMC promoted the health of LBW newborn.

Mothers, irrespective of the selected wrap, appeared to recommend the new wrap over the traditional one. Even those mothers choosing the traditional wrap reported that it would have been easier for them and their families to practice KMC at home if they had chosen the new wrap. They also mentioned that they would strongly recommend the new wrap to other mothers, as they do not want other mothers to experience the problems they confronted using traditional wrap. The mothers using new wrap reported the major benefit of the new wrap was that they could provide KMC alone as the wrap could be tied without anybody’s help.

*“Even when there’s no one at home*, *the new wrap is of great help since I can tie it on my own”*.

(New wrap user)

On the contrary, mothers using traditional wrap reported that the traditional wrap was cumbersome in providing continuous KMC as they always needed extra person throughout the day to tie and untie the knot to keep the newborn in KMC, after taking out for breastfeeding, and changing diapers.

“*I had to go through great struggle to practice KMC at home as I always needed someone to help me tie the wrap*.*”*

(Traditional wrap user)

The other benefit offered by the new wrap was about the security. Mothers reported that they felt secure while moving around practicing KMC with the new wrap as it was tied securely with belt. All mothers choosing new wrap performed regular household chores such as cooking, washing and drying cloths, sweeping the house etc. One mother who was a new wrap user reported, “*New wrap is extremely convenient*, *not only did I practice KMC at home*, *but I also continued it while looking after the goats in the farm after 22 days of delivery*”.

On the contrary, none of the mothers using the traditional wrap performed household chores while practicing KMC as they were not comfortable with the security offered by the wrap especially in conditions when they had to tie the wrap themselves or when they had to seek help from untrained persons.

“*Family member trained in the hospital for tying the traditional wrap is not always available at home*, *and when I seek help from other untrained relatives/neighbors*, *they cannot tie the wrap securely*”.

(Traditional wrap user)

Mothers using the new wrap reported that it was easy to breastfeed during KMC as she could easily slacken the strap herself for feeding. With the new wrap, it was easy to rest on the bed or chair while practicing KMC. They also reported that with the new wrap it was easier to get help from male members of the family, since the new wrap was perceived as a baby carrier by male members which led to their engagement in keeping the newborn in KMC. Husbands of new wrap users found it was easy to fasten, and considered it safe.

“*During the hospital stay my husband preferred to carry the newborn in new wrap as he felt less embarrassed with it*. *However as I chose the traditional wrap*, *he avoided keeping the newborn in KMC at home*.*”*

(Traditional wrap user)

During the interview, mothers were inquired if they would have purchased the wrap if it were not provided free of cost and the amount they were willing to pay if they agreed to pay. All interviewed mothers reported that they would have purchased the wrap for KMC even if it had not been provided free of cost, as the wrap facilitated KMC which had positive effect on the health of their babies and it was comfortable to use. The price reported by mother ranged from 1 to 10 dollar for new wrap and from 5 to 12 dollars for the traditional wrap.

## Discussion

Despite KMC being introduced in Nepal over a decade ago [16] and being incorporated in various in service and pre-service curricula of health workers, it is still not standard practice in most facilities. The present study attempts to assess the feasibility of a KMC with the provision of new wrap as an intervention to improve the adoption of KMC by improving the experience related to therapy.

Several systematic reviews have explored the enablers and barriers of KMC [9–11]. Lack of comprehensive information to mothers about benefits of KMC, shortage of time needed to provide KMC due to household obligations, low motivation among mothers, support from family members as result of pain/fatigue associated with practicing KMC for extended period are some of the major barriers reported [9–11].

Majority of mothers included in our study were from Dalit, Janajati and Madeshi ethnic group. A study analyzing data from NDHS 2011 reported the prevalence of LBW was equal among all ethnic groups [17]. Another study from Tertiary hospital in Kathmandu reported that majority of mothers with LBW newborns belonged to advantaged ethnicity including Brhamin, Chhetri and advantaged Janajati [18]. The higher number of Dalit, Janajati and Madeshi in our study population might be because of the study hospital caters mostly to these ethnicity.

In our study, a majority of the mothers practiced STS contact for nearly about 24 hours during hospital stay irrespective of the selected wrap. A longitudinal study from Ghana with 202 mothers of LBW newborns reported only 18% of the continuous KMC during hospital stay [19], while a study from Bangladesh with 423 LBW newborns reported that mean duration of STS contact was only 10.7 hours during hospital stay for stable LBW newborn groups [20]. The higher rate of STS during hospital stay in our study might be due to increased capacity of health workers to train mothers and families on KMC. Health workers reported that they were not aware about KMC as a method of care for LBW newborns, and none of them had counseled mothers of LBW newborn for KMC practice prior to the study. However, following the training, they were more confident in the management of LBW newborns through KMC. Studies have reported that limited knowledge of health worker about KMC and lack of belief in its outcome as a major barrier for KMC uptake [9, 21]. Other very important factors that contributed to KMC adherence at hospital include: a) improved understanding about the danger signs of LBW and benefits of KMC among mothers and families due to counseling; 2) involvement of the mothers in the decision on the choice of wrap; and 3) demonstration of KMC and the opportunity to practice with anatomical models before actually practicing with their small babies. A review on barriers and enablers of KMC practice have reported that increased understanding of mothers positively influence the adherence to KMC [11].

No significant difference was observed in the hours of STS contact among mothers using the traditional wrap and the new wrap during hospital stay. This might be because mothers from both group received the same level of training, encouragement and support by health workers at the postnatal ward. An additional reason might be that household obligations which are identified as a major barrier for KMC did not exist in the hospital. Studies have reported that there was more time for KMC at hospital than at home due to everyday household tasks burdened on women [11, 22].

It is essential to assure that the mothers initiating KMC in the hospitals continue it at home after discharge from the hospital to ensure optimal benefit. Most of the mothers in our study discharged from the hospital expressed an intention to continue KMC at home. This finding is consistent with the study done in Ghana where 95.5% of the mothers were willing to continue KMC at home [19]. Almost all women who attended the 28 day postnatal visit reported continuing KMC at home. A descriptive study in tertiary care teaching hospital in India reported that 82.5% of the mothers continued with STS contact 45 days post discharge [23]. The higher rate of KMC adherence at home in our study might be the result of mothers and families’ engagement in decision-making regarding KMC, choice of wrap ensuring family support to practice KMC.

Mothers using the new wrap appeared to report more hours of STS practiced at home compared to the traditional wrap. This could be attributed to some of the benefits provided by the characteristics of the new wrap as ease of wearing the wrap, providing security while holding the newborn and able to perform household chores. Several studies have identified that the time required for providing KMC as a major barrier for caregivers, including mothers, due to their obligations at home, and lack of support inhibits them from providing the time required to provide continuous KMC [9–10]. Mothers reported that with the new wrap it was convenient for them to practice KMC at home continuously, as it allowed the security and flexibility to perform household chores while practicing KMC, and promoted division of labor in KMC, as male members of the family wore the new wrap. Studies have reported that the involvement of fathers positively affected KMC uptake through division of labor or by making the mother feel comfortable [10, 24].

Several studies have reported that practicing KMC promotes breastfeeding [19, 25]. All mothers in our study exclusively breastfed their LBW newborns during hospital stay and most continued to do so after discharge. Breastfeeding is reportedly easier with the new wrap since the mother can slacken the knot of the wrap herself and feed the without help of another person.

### Strength and limitation

The strength of our study is the use of a mixed methods approach to assess the feasibility of introducing a new wrap. Courtesy bias for qualitative data collection was minimized by hiring a qualitative researcher who was not part of the study team.

A key limitation of the study is the small sample size in one group as compared with the other. The number of mothers choosing traditional wrap was quite low, creating an imbalance in the group numbers and further reducing the power to detect a difference in total hours of STS.

Information on hours of STS contact practiced by mother and exclusive breastfeeding at home was captured by phone interviews rather than direct observations. This has the potential for both recall bias and social desirability bias. The study was not powered to evaluate the differences in the hours of STS practiced, or impact on neonatal mortality.

## Conclusions

Involvement of mothers with the provision of a new ergonomic wrap of their choice led to high acceptability, uptake and continuation of KMC practice during hospital stay and at home. This new wrap may have made a difference in KMC continuation rate at home as the new wrap was designed in a way that it securely holds the newborn, allows flexibility for performing household chores while practicing KMC and acceptable for use by male members of the family. Recently with increased attention and investment with Every Newborn Action Plan (ENAP), KMC has been highly prioritized through NENAP where government of Nepal aims to have 35% of all premature newborns receive KMC by 2035 from a present situation of 0% coverage. The involvement of mothers and families with provision of new ergonomic wrap, is very timely and can be taken up by the government of Nepal while scaling up the KMC practice.

## Supporting information

S1 Fig Picture showing use of traditional wrap for KMC. (TIFF)

S2 Fig Picture showing use of new wrap for KMC. (TIFF)

S3 Fig Interview guide–English version. (DOCX)

S4 Fig Interview guide–Nepali version. (DOCX)

S5 Fig Topic guide–English version. (DOCX)

S6 Fig Topic guide–Nepali version. (DOCX)

S7 Fig Study questionnaire–English version. (DOCX)

S8 Fig Study questionnaire–Nepali version. (DOCX)
