# Supplementary figures and images for: Feasibility assessment of an ergonomic baby wrap for kangaroo mother care: A mixed methods study from Nepal
Source: PLoS One. 2018 Nov 15;13(11):e0207206. doi: 10.1371/journal.pone.0207206 (PMC6237334; doi:10.1371/journal.pone.0207206)

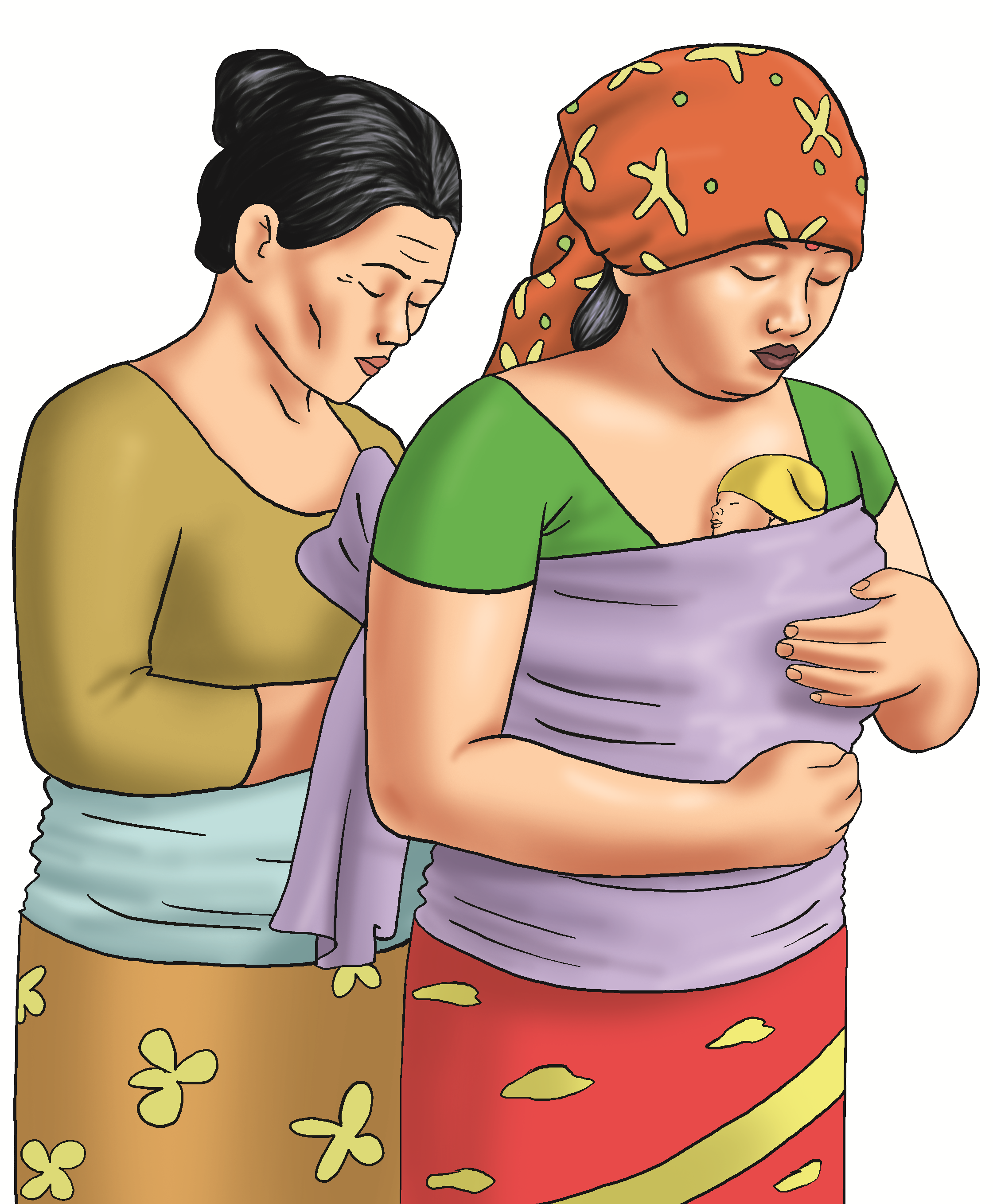

Supplement: S1 Fig — (TIFF) [file pone.0207206.s001.tiff]

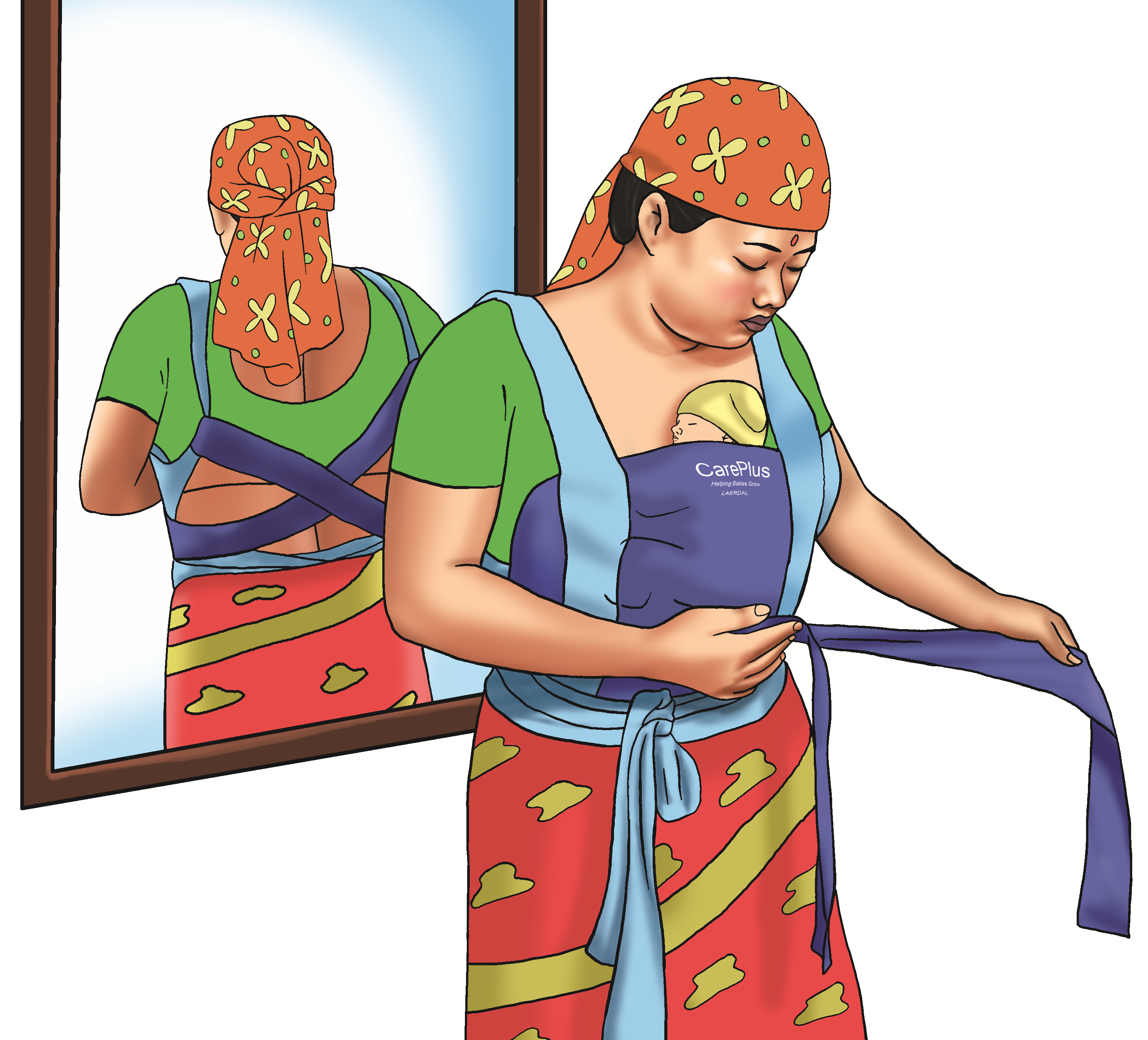

Supplement: S2 Fig — (TIFF) [file pone.0207206.s002.tiff]
