# Supplementary material for: Feasibility assessment of an ergonomic baby wrap for kangaroo mother care: A mixed methods study from Nepal
Source: PLoS One. 2018 Nov 15;13(11):e0207206. doi: 10.1371/journal.pone.0207206 (PMC6237334; doi:10.1371/journal.pone.0207206)
Supplement: S3 Fig — (DOCX) [file pone.0207206.s003.docx]

**Promoting Kangaroo Mother Care in Selected Hospitals of Nepal through Training and Provision of Baby Wrap**

**In-depth interview guideline for selected study participant (recently delivered mothers of stable preterm/LBW babies)**

Objective: Explore the rate of KMC continuation and compare opinion about two wraps.

**Background information**

**Date of interview**:----------------------- **District**:----------------------

**Health Facility**:------- **Municipality/VDC**:---------- **Ward No**:--------

**Name of the respondent**:------------------------------- **Type of wrap chosen**: :---------------------

**Level of KMC usage**: ----------------------------

1. Your baby was born smaller or earlier than usual. How has the baby’s health been since birth?
2. What kinds of special practices have you used since your baby is smaller than usual?
   1. Probe for: experiences related to KMC; benefits, drawbacks, etc.
   2. Probe for: breastfeeding, if not specifically mentioned.
3. After your baby was born, you were given the chance to try two different wraps for carrying the baby. You chose XXX wrap. What factors led you to choose this wrap?
4. How have you been using the wrap during the past month?
5. What does your husband think about the wrap (i.e. what’s his opinion)?

Probe: Has he used it for carrying the baby?

1. What do other family members in your household think about the wrap (i.e. what’s their opinion of it)?

Probe: Has this person used the wrap for carrying the baby**.**

1. What are the good aspects of this wrap, in your opinion?
2. What could be improved about the wrap?

**For mothers with low KMC usage only:** Can you please elaborate the reason behind for not practicing KMC as recommended by the health workers?

1. **Perception about wrap chosen**

- How comfortable were you about the security of baby while practicing KMC with the chosen wrap? (probe reason for both positive and negative answer).
- How easy was it to practice KMC for you with the chosen wrap at home? Please provide with reasons.
- How was the perception of other family members (husband, parents in law) about the chosen wrap?

1. **Benefits of practicing KMC (to parents and to babies)**

- Was KMC beneficial for you baby? If yes, can you please elaborate how it was beneficial, or in what ways was it beneficial? If no, can you please elaborate how?
- Was KMC beneficial to you as parents? If yes, can you please elaborate how it was beneficial, or in what ways was it beneficial? If no, can you please elaborate how?

**For mothers with low KMC usage only:** if the answers to the above questions are yes, please specify the reasons for not practicing KMC at home despite being aware about its usefulness.

1. **Recommendation**

- Do you recommend KMC practice to other mothers with preterm/LBW babies?
- Do you recommend using the same wrap you have chosen while practicing KMC?
