# Supplementary material for: Feasibility assessment of an ergonomic baby wrap for kangaroo mother care: A mixed methods study from Nepal
Source: PLoS One. 2018 Nov 15;13(11):e0207206. doi: 10.1371/journal.pone.0207206 (PMC6237334; doi:10.1371/journal.pone.0207206)
Supplement: S4 Fig — (DOCX) [file pone.0207206.s004.docx]

**tflnd tyf jRrfnfO{ Gofgf] kfg]{ sk8fsf] (baby wrap) dfWod4f/f (KMC) ljlwnfO{ k|f]T;fxg ug]{af/] cWoog**

**5flgPsf cWoog ;xefuLsf nflu cGtjf{tf{ lgb]{lzsf -dlxgf gk'uL÷tf}{n sd eP/ hlGdPsf l:y/ :jf:Yo ePsf aRrfx?sf] cfdfnfO{_**

p¢]]Zo M dfofsf] c+ufnf]sf] lg/Gt/tfsf] b/ af/]df cg';Gwfg / lzz'nfO{ Gofgf] kfg{] b'O{vfn] sk8fsf] ;DaGwdf ePsf wf/0ffx?sf] t'ngf

k[i7e"ld

cGt{jftf{ lnPsf] ldlt=========================================== lhNnf===================================================

:jf:Yo ;+:yf======================================= gu/kflnsf÷uf=lj=;============================ jf8{ g+==========

pQ/bftfsf] gfd============================================== /f]h]sf] Gofgf] kfg{] sk8f=====================================

dfofsf] c+ufnf]sf] k|of]usf]] tx======================================================================================

!= tkfO{sf] aRrf ;fdfGo cj:yfeGbf ;fgf] cyjf rf8} hlGdPsf] lyof] < aRrf hlGdP b]lv aRrfsf] :jf:Yo s:tf] 5 <

@= tkfO{sf] aRrf c? aRrfeGbf ;fgf] ePsfn] tkfO{n] s:tf] lsl;dsf] laz]if cEof; ul//xg' ePsf] 5 <

# s­_ k|f]j ug]{ M dfofsf] c+ufnf] ;DalGw cg'ej, kmfObfx?, a]kmfObfx? cflb

v_ k|f]j ug]{ M :tgkfg, pNn]v gu/]sf laz]if s'/fx?

#= tkfO{sf] aRrf hGd]kl5 tkfO{nfO{ aRrf af]Sg b'O{ leGg lsl;dsf] aRrfnfO{ Gofof] kfg{] sk8f /f]Hg] cj;/ lbOPsf] lyof], tkfO{n] XXX Gofgf] kfg{] sk8f /f]Hg' ePsf] lyof] . tkfO{n] s] sf/0fn] ubf{ Tof] sk8f /f]Hg' ePsf] lyof] <

$= tkfO{n] ut dlxgfb]lv s;/L of] aRrf Gofgf] kfg{] sk8f k|of]u ul//xg'ePsf] 5 <

%= aRrf Gofgf] kfg{] sk8fsf] af/]df tkfO{sf] >Ldfgsf] s] wf/0ff 5 <

k|f]j ug]{ M s] tkfO{sf] >Ldfgn] aRrf af]Sg of] sk8fsf] k|of]u ug{'ePsf] 5 <

^= aRrf Gofgf] kfg{] sk8fsf] af/]df tkfO{sf] 3/sf cGo ;b:ox?sf] s] wf/0ff 5 <

k|f]j ug]{ M s] tkfO{sf]] 3/sf cGo ;b:ox?n] aRrf af]Sg of] sk8fsf] k|of]u ug{'ePsf] 5 <

&= tkfO{sf] larf/df aRrf Gofgf] kfg{] sk8fsf] /fd|f] kIfx? s] s] x'g\ <

*= aRrf Gofgf] kfg{] sk8fdf s] ;'wf/ ug{ ;lsG5 <

**dfofsf] c+ufnf]sf] cEof; sd ug{] cfdfx?sf nflu dfq M** :jf:YosdL{x?n] l;kmfl/; u/]sf] dfofsf] c+ufnf]sf] cEof; tkfO{n] gug{'sf] sf/0f s[kof la:tf/df atfO{lbg'xf];\ .

(= **/f]Hg' ePsf] aRrf Gofgf] kfg{] sk8f ;DalGw wf/0ff**

- - tkfO{n] /f]Hg' ePsf] aRrf Gofgf] kfg{] sk8fn] dfofsf] c+ufnf]sf] cEof; ubf{ tkfOn]{ aRrfsf] ;'/Iffsf] af/]df slQsf] ;xh dx;'; ug'{eof]] <-;sf/fTds / gs/fTds sf/0f k|f]j ug{]_
  - tkfO{n] /f]Hg' ePsf] aRrf Gofgf] kfg{] sk8fn] 3/df dfofsf] c+ufnf]sf] cEof; ug{ tkfO{nfO{ slQsf] ;lhnf] ePsf] lyof] < s[kof sf/0f ;lxt atfO{lbg'xf];\ .
  - tkfO{n] /f]Hg'ePsf] aRrf Gofgf] kfg{] sk8fsf] af/]df tkfO{sf] kl/jf/sf] cGo ;b:ox?sf] ->Ldfg, ;f;';;'/f_ s] wf/0ff 5 <

!)= **dfofsf] c+ufnf]sf] cEof; ug{'sf kmfO{bfx? -cfdfafa' / aRrfnfO{_**

- - s] tkfO{sf] aRrfnfO{ dfofsf] c+ufnf]sf] cEof; kmfObfhgs lyof] < olb kmfObfhgs lyof] eg] s;/L / s'g tl/sfn] kmfObfhgs lyof] la:tf/ ul/lbg'xf];\ . lyPg eg] s;/L lyPg la:tf/ ul/lbg'xf];\ .
  - s] tkfO{nfO{ cfdfsf] ?kdf÷x}l;otn] dfofsf] c+ufnf] kmfObfhgs lyof] < olb kmfObfhgs lyof] eg] s;/L / s'g tl/sfn] kmfObfhgs lyof] la:tf/ ul/lbg'xf];\ . lyPg eg] s;/L lyPg la:tf/ ul/lbg'xf];\ .

**dfofsf] c+ufnf]sf] cEof; sd ug{] cfdfx?sf nflu dfq M**

dflysf k|Zgx?sf pQ/ ;a} 7Ls x'g eg] dfofsf] c+ufnf]sf] kmfObfx?sf] hfgsf/L x'bfFx'b} klg tkfO{n] 3/df o;sf] cEof; gug{'sf] sf/0fx? s] s]] x'g\ <

**!!= l;kmfl/;**

- - s] tkfO{ dlxgf gk'uL hGd]sf÷sd tf}n hGd]sf aRrfx?sf cfdfx?nfO{ dfofsf] c+ufnf]sf] cEof; ug{ l;kmfl/; ug{'x'G5 <
  - s] tkfO{ c? cfdfx?nfO{ dfofsf] c+ufnf]sf] cEof; ubf{ tkfO{n] /f]Hg'ePsf] aRrf Gofgf] kfg{] sk8f k|of]u ug{ ;'emfa lbg'x'G5 <
