# Supplementary material for: Feasibility assessment of an ergonomic baby wrap for kangaroo mother care: A mixed methods study from Nepal
Source: PLoS One. 2018 Nov 15;13(11):e0207206. doi: 10.1371/journal.pone.0207206 (PMC6237334; doi:10.1371/journal.pone.0207206)
Supplement: S5 Fig — (DOCX) [file pone.0207206.s005.docx]

**Promoting Kangaroo Mother Care in Selected Hospitals of Nepal through Training and Provision of Baby Wrap**

**Topic guide for FGD with health workers**

**Objective: Explore perception of and understanding of KMC after the training and compare opinions about two wraps**

**Date of FGD**:-----------------------

**Health Facility**: Amda Hospital

Koshi Zonal Hospital

1. **Perception and Understanding of KMC before and after training**

Probing questions

- What were you perception about KMC therapy before training?
- Did your perceptions and understanding about KMC therapy change after training? If yes, can you please specify in what ways?
- Were you practicing KMC therapy before training? If yes, can you please share your experiences about facilitating KMC before and after training?
  - If no, can you please explain why were you not practicing KMC before training?

1. **Opinion about Care plus wrap compared to traditional wrap**

**Can you please elaborate opinion about traditional wrap compared to Care Plus wrap?**

Probing questions: Compare opinion in terms of:

- Perceived security in terms of holding baby
- Comfortable for the parents
- Facilitates breastfeeding
- Facilitates performing household cores
- Challenges observed among mothers using care plus wrap and traditional wrap.
- Gender neutral (acceptable to use for fathers)
- Others (specify)………………………………………………………………………….

1. **Recommendation**

As health care workers, if you are to recommend KMC for mothers after the study period or outside study facility, which wrap will you recommend and why?
