# Supplementary material for: Feasibility assessment of an ergonomic baby wrap for kangaroo mother care: A mixed methods study from Nepal
Source: PLoS One. 2018 Nov 15;13(11):e0207206. doi: 10.1371/journal.pone.0207206 (PMC6237334; doi:10.1371/journal.pone.0207206)
Supplement: S6 Fig — (DOCX) [file pone.0207206.s006.docx]

**tflnd tyf jRrfnfO{ Gofgf] kfg]{ sk8fsf] (baby wrap) dfWod4f/f (KMC) ljlwnfO{ k|f]T;fxg ug]{af/] cWoog**

**:jf:Yo sfo{stf{;Fu ul/g] ;d"x s]Gb|Lt 5nkmnsf] nflu lgb]{lzsf**

**p2]ZoM** tflnd k|Zrft dfofsf] c+ufnf]sf] af/]df a'emfO{ / aRrfnfO{ Gofgf] kfg]{ b'O{ vfn] sk8f af/]df t'ngfTds ljrf/sf] vf]hL ug]{

5nkmnsf] ldltM **====================================================**

:jf:Yo ;+:yfM cfDbf c:ktfn

sf]zL c+rn c:ktfn

**!= tflnd cl3 / kl5 dfofsf] c+ufnf]sf] wf/0ff / a'emfO**

k|f]j ug]{ k|Zgx?

- tflnd cl3 dfofsf] c+ufnf]af6 ul/g] pkrf/sf] af/]df tkfO{sf] s:tf] wf/0ff lyof] <
- s] tkfO{sf] tflndkl5 dfofsf] c+ufnf]af6 ul/g] pkrf/sf] af/]df wf/0ff / a'emfOdf kl/jt{g cfof] < olb cfof] eg] s]–s] df kl/jt{g cfof] atfOlbg ;Sg'x'G5 <
- s] tkfO{n] tflnd cl3 dfofsf] c+ufnf]af6 ul/g] pkrf/sf] cEof; ul//xg' ePsf] lyof] < olb lyof] eg] tflnd cl3 / kl5 dfofsf] c+ufnf]sf] ;xlhs/0f af/]df tkfO{sf] cg'ej atfpg'xf];\ .
  - olb lyPg eg] tflnd cl3 dfofsf] c+ufnf] ;DalGw cEof; lsg ug'{ePg atfOlbg ;Sg'x'G5 <

**@= k/Dk/fut ?kdf k|of]u u/L cfPsf] Gofgf] kfg]{ sk8fsf] t'ngfdf s]o/ Kn; Gofgf] kfg]{ sk8f af/]df ljrf/**

k/Dk/fut ?kdf k|of]u u/L cfPsf] Gofgf] kfg]{ sk8fsf] t'ngfdf s]o/ Kn; Gofgf] kfg]{ sk8fsf] af/]df tkfO{sf] ljrf/ lj:t[t ?kdf atfO{lbg' x'G5 <

k|f]j ug]{ k|Zgx? M ljrf/sf] t'ngf ug]{ M

- aRrfnfO{ ;dfpFbf ;'/Iff eP gePsf] af/]df wf/0ff
- cleefassf] nflu ;'ljwfhgs
- :tgkfgsf] ;xhLs/0f
- 3/fo;L sfdsfh ug{ ;xhLs/0f
- k/Dk/fut Gofgf] kfg]{ sk8f / s]o/ Kn; Gofgf] kfg]{ sk8f k|of]u ul//x]sf cfdfx?df canf]sg ul/Psf r'gf}tLx?
- n}+lus t6:ytf -afa'x?sf] :jLsfo{tf_
- cGo -pNn]v ug]{_ ==================================================================

**#= l;kmfl/;**

:jf:Yo sfo{stf{sf] gftfn] olb tkfO{n] of] cWoog cjlwkl5 jf of] cWoog ;+:yfeGbf aflx/ dfofsf] c+ufnf] af/]df ;'emfj lbg' k/]df tkfO{ s'g Gofgf] kfg]{ sk8fsf] l;kmfl/; ug'{x'G5 / lsg <
