# Supplementary material for: Feasibility assessment of an ergonomic baby wrap for kangaroo mother care: A mixed methods study from Nepal
Source: PLoS One. 2018 Nov 15;13(11):e0207206. doi: 10.1371/journal.pone.0207206 (PMC6237334; doi:10.1371/journal.pone.0207206)
Supplement: S7 Fig — (DOCX) [file pone.0207206.s007.docx]

| **Promoting Kangaroo Mother Care in Selected Hospitals of Nepal through Training and Provision of Baby Wrap** | | |
| --- | --- | --- |
| **To be filled by Jhpiego staff with mother of LBW/Preterm babies during stay at health facility, during phone survey and PNC follow up** | | |
| **Section A: To be filled at the time of discharge from health facility** | | |
| 1. **Background information** | | |
| - 1. District | - 1. Hospital | |
| - 1. IP no | - 1. ID no. | |
| - 1. Ethnicity^[[1]](#footnote-1)^ | - 1. Educational status^[[2]](#footnote-2)^ | |
| - 1. Municipality/VDCs | - 1. Ward no | |
| - 1. Age (completed yrs) | - 1. Parity: | |
| - 1. Date of Delivery | - 1. Gestation week | |
| - 1. Type of Delivery | Spontaneous Vaginal | |
|  | Forcep | |
|  | Vacuum | |
| - 1. Birth weight ( grams) | - 1. Sex : | Male |
|  |  | Female |
| - 1. Contact number: | | |

| 1. **KMC related information** | |
| --- | --- |
| 1. Date of KMC admission | 1. Time of KMC admission |

| 1. Sequence of random allocation | | |
| --- | --- | --- |
| First wrap | Time started | Time ended |
| Second wrap | Time started | Time ended |

| 1. Wrap chosen after twelve hours: | Traditional |
| --- | --- |
|  | Care Plus |

| 1. Reason for Choosing the particular **wrap** | | | |
| --- | --- | --- | --- |
| **Variables** | **Agree** | **Unsure** | **Disagree** |
| Safe and Secure |  |  |  |
| Allows movement |  |  |  |
| Less fatigue |  |  |  |
| Easy to use |  |  |  |
| Facilitates breastfeeding |  |  |  |
| Easy to monitor baby condition |  |  |  |
| Gender neutral  (Design of wrap is acceptable for fathers or male members.) |  |  |  |
| Other (specify)…………………………………………………………………………….. | | | |
| 1. Important perceptions of the mother about selected wrap (*reason why she selected particular wrap*)   ……………………………………………………………………………………….….……………………………………………………………………………………………………………………………………………………………………………………………………………… | | | |
| 1. Important experiences of the mother about selected wrap (*any difficulties faced while practicing KMC*?)   ……………………………………………………………………………………….….……………………………………………………………………………………………………………………………………………………………………………………………………………… | | | |

| 1. Type of KMC practiced during stay at health facility (please mention the duration in hours) | **Days** | **Hours** | **Discontinued**  *(go to question 2.9)* |
| --- | --- | --- | --- |
|  | Day 1 |  |  |
|  | Day 2 |  |  |
|  | Day 3 |  |  |
|  | Day 4 |  |  |
|  | Day 5 |  |  |
|  | Day 6 |  |  |
|  | Day 7 |  |  |
|  | Day 8 |  |  |
|  | Day 9 |  |  |
|  | Day 10 |  |  |

| 1. Can you please provide the reason for discontinuation?   ***(multiple answers )*** | Physical pain following labor |  |
| --- | --- | --- |
|  | Fear of harming baby through cord stump |  |
|  | Fear of baby falling |  |
|  | Discomfort |  |
|  | Lack confident |  |
|  | Lack of time |  |
|  | Not beneficial |  |
|  | Felt tired |  |
|  | Lack of support from family |  |
|  | Social stigma |  |
|  | Baby doesnot want to be in KMC position |  |
|  | Healthy baby |  |
|  | Others(Specify)……… …..…………..…… ……… | |

| 1. Weight of baby at time of discharge (grm) |
| --- |
| 1. Health workers observation (*any important observation regarding use of the wrap which was not* chosen *by mother with respect to reason for not choosing and practice with on this wrap*.)   ……………………………………………………………………………………………………………………………………………………………………………………………………………………………………………………………………………………………… |

| **Section B: To be filled during phone survey (after 24 hours and within 48 hours of discharge)** | | | | | | | |
| --- | --- | --- | --- | --- | --- | --- | --- |
| Hello!! Namaskar, My name is …………………..and I am calling from ……………………………………………. May I please speak to ………..? How are you? How is your baby? As you know, we are conducting a study on behalf of recently delivered mothers with stable LBW/preterm babies to explore adherence to KMC practice. You indicated your willingness to participate in the study during your delivery at ……..hospital. In continuation of the study I am calling you to discuss about KMC practices at your home. May I take your few minutes to ask few questions related to this? Thank you. | | | | | | | |
| 1. **Background Information** | | | | | | | |
| - 1. ID no. | | | - 1. Date of Follow up | | | | |
| - 1. **Status of follow up** | | | | | | | |
| 1.3.1. Able to Contact | | | 1.3.2. Not able to contact  (If not able to contact while contacting three times per day for three consecutive days) | | | | |
| Reason for not being able to contact:……………………………………………….. | | | | | | | |
| **Follow up questions** | | | | | | | |
| 1. **Condition of mother and baby** | | | | | | | |
| - 1. How are you? | Fine | | | |  | | **2**  2.3 |
|  | Not feeling well | | | |  | | |
| - 1. What are the health problems?   (multiple response) | Fever | | | |  | | |
|  | Pain in wound | | | |  | | |
|  | Breast engorgement | | | |  | | |
|  | Loos motion | | | |  | | |
|  | Vomiting | | | |  | | |
|  | Cough and congestion | | | |  | | |
|  | Others specify………………………………………… | | | | | | |
| - 1. How is your baby | Fine | | | |  | 2.5 | |
|  | Not feeling well | | | |  | | |
| - 1. What are the problems | Feeding problem | | | |  | | |
|  | Sleeping problem | | | |  | | |
|  | Fever | | | |  | | |
|  | Loose motion | | | |  | | |
|  | Vomiting | | | |  | | |
|  | Cough and Congestion | | | |  | | |
|  | Others specify………………………………………… | | | | | | |
| - 1. Are you exclusively breastfeeding your baby? | Yes | | | No | | | |
| 1. **KMC practice** | | | | | | | |
| - 1. Are you continuing KMC at home? | | Yes | | | No  3.5 | | |
| - 1. How many hours did you perform KMC in last 24 hours? | |  | | | | | |

| - 1. Are you practicing KMC during day time while performing household chores? | Yes | No |
| --- | --- | --- |
| - 1. Are you practicing KMC at night? | Yes | No |

| - 1. Can you please provide the reason for discontinuation?   **(Multiple answers)** | Physical pain following labor |  |
| --- | --- | --- |
|  | Fear of harming baby through cord stump |  |
|  | Fear of baby falling |  |
|  | Discomfort |  |
|  | Lack confident |  |
|  | Lack of time |  |
|  | Not beneficial |  |
|  | Felt tired |  |
|  | Lack of support from family |  |
|  | Social stigma |  |
|  | Baby doesnot want to be in KMC position |  |
|  | Healhty baby |  |
|  | Others (Specify)……… …..…………..…… ……… ………………… …….. | |

| 1. **Perception of KMC wrap** | **Agree** | **Unsure** | **Disagree** |
| --- | --- | --- | --- |
| - 1. It is comfortable to perform KMC with chosen wrap. |  |  |  |
| - 1. The chosen wrap can hold the baby securely during KMC. |  |  |  |
| 1. **Benefits of KMC** | | | |

| - 1. What are the benefits of KMC?   **(multiple response)** | Facilitates breastfeeding | |  |  |
| --- | --- | --- | --- | --- |
|  | Provides natural warmth to the baby | | |  |
|  | Reduce infection for baby | |  |  |
|  | Promotes weight gain | | |  |
|  | Easy to monitor condition of baby | |  |  |
|  | Facilitates mother and child bonding | | |  |
|  | Others (specify)…………………………… | | | |
| 1. **Spousal and Family Acceptability** | | | | |
| - 1. Is your husband also providing KMC?   (doing KMC during your time for dinner, toilet, bathing, rest etc) | Yes | No | NA | |
| - 1. Is your family member also providing KMC?   (doing KMC during your time for dinner, toilet, bathing, rest etc) | Yes | No | NA | |

| **Section C: To be filled during phone survey (week 1)** | | | | | |
| --- | --- | --- | --- | --- | --- |
| Hello!! Namaskar, My name is …………………..and I am calling from ……………………………………………. May I please speak to ………..? How are you? How is your baby? As you know, we are conducting a study on behalf of recently delivered mothers with stable LBW/preterm babies to explore adherence to KMC practice. You indicated your willingness to participate in the study during your delivery at ……..hospital. In continuation of the study I am calling you to discuss about KMC practices at your home. May I take your few minutes to ask few questions related to this? Thank you. | | | | | |
| 1. **Background Information** | | | | | |
| - 1. ID no. | | - 1. Date of Follow up | | | |
| - 1. **Status of follow up** | | | | | |
| 1.3.1. Able to Contact | | 1.3.2. Not able to contact  (If not able to contact while contacting three times per day for three consecutive days) | | | |
| Reason for not being able to contact:……………………………………………….. | | | | | |
| **Follow up questions** | | | | | |
| 1. **Condition of mother and baby** | | | | | |
| - 1. How are you? | Fine | |  | | **2.3** |
|  | Not feeling well | |  | | |
| - 1. What are the health problems?   (multiple response) | Fever | |  | | |
|  | Pain in wound | |  | | |
|  | Breast engorgement | |  | | |
|  | Loos motion | |  | | |
|  | Vomiting | |  | | |
|  | Cough and congestion | |  | | |
|  | Others specify………………………………………… | | | | |
| - 1. How is your baby | Fine | | | 2.5 | |
|  | Not feeling well | | |  | |
| - 1. What are the problems | Feeding problem | | |  | |
|  | Sleeping problem | | |  | |
|  | Fever | | |  | |
|  | Loose motion | | |  | |
|  | Vomiting | | |  | |
|  | Cough and Congestion | | |  | |
|  | Others specify………………………………………… | | | | |
| - 1. Are you exclusively breastfeeding your baby? | Yes | | | No | |

| 1. **KMC practice** | | |
| --- | --- | --- |
| - 1. Are you continuing KMC at home? | Yes | No |
| - 1. How many hours do you practice KMC per day (including both day and night) in this week. | Dates | Hours |
|  |  |  |
|  |  |  |
|  |  |  |

| **Section D: To be filled during phone survey (week 2)** | | | | | | | |
| --- | --- | --- | --- | --- | --- | --- | --- |
| Hello!! Namaskar, My name is …………………..and I am calling from ……………………………………………. May I please speak to ………..? How are you and your baby? As you know, we are conducting a study on behalf of recently delivered mothers with stable preterm/LBW to explore adherence to KMC practice. As talked with you last week over the telephone. I am calling you to discuss about KMC practices at your home as continuation of the study. May I take your few minutes to ask few questions related to this? Thank you. | | | | | | | |
| 1. **Background Information** | | | | | | | |
| - 1. ID no. | | | - 1. Date of Follow up | | | | |
| - 1. **Status of follow up** | | | | | | | |
| 1.3.1 Able to Contact | | | 1.3.2 Not able to contact  (If not able to contact while contacting three times per day for three consecutive days) | | | | |
| Reason for not being able to contact:……………………………………………….. | | | | | | | |
| **Follow up questions** | | | | | | | |
| 1. **Condition of mother and baby** | | | | | | | |
| - 1. How are you? | | Fine | | | |  | **2.3** |
|  |  | Not feeling well | | | |  | |
| - 1. What are the health problems?   (multiple response) | | Fever | | | |  | |
|  |  | Pain in wound | | | |  | |
|  |  | Breast engorgement | | | |  | |
|  |  | Loos motion | | | |  | |
|  |  | Vomiting | | | |  | |
|  |  | Cough and congestion | | | |  | |
|  |  | Others specify………………………………………… | | | | | |
| - 1. How is your baby | | Fine | | | |  | 2.5 |
|  |  | Not feeling well | | | |  | |
| - 1. What are the problems | | Feeding problem | | | |  | |
|  |  | Sleeping problem | | | |  | |
|  |  | Fever | | | |  | |
|  |  | Loose motion | | | |  | |
|  |  | Vomiting | | | |  | |
|  |  | Cough and Congestion | | | |  | |
|  |  | Others specify………………………………………… | | | | | |
| - 1. Are you exclusively breastfeeding your baby? | | Yes | | | No  3.5 | | |
| 1. **KMC practice** | | | | | | | |
| - 1. Are you continuing KMC at home? | Yes | | | No | | | |

| - 1. How many hours do you practice KMC per day (including both day and night) in this week. | **Dates** | **hours** |
| --- | --- | --- |
|  |  |  |
|  |  |  |
|  |  |  |
|  |  |  |
|  |  |  |
|  |  |  |
|  |  |  |

| - 1. Are you practicing KMC in day time while performing household chores? | | | | | | Yes | | | | | | No | | | | |
| --- | --- | --- | --- | --- | --- | --- | --- | --- | --- | --- | --- | --- | --- | --- | --- | --- |
| - 1. Are you practicing KMC at night? | | | | | | Yes | | | | | | No | | | | |
| - 1. Can you please provide the reason for discontinuation?   ***(multiple answers )*** | | | | Physical pain following labor | | | | | | | | | | |  | |
|  |  |  |  | Fear of harming baby through cord stump | | | | | | | | | | |  | |
|  |  |  |  | Fear of baby falling | | | | | | | | | | |  | |
|  |  |  |  | Discomfort | | | | | | | | | | |  | |
|  |  |  |  | Lack confident | | | | | | | | | | |  | |
|  |  |  |  | Lack of time | | | | | | | | | | |  | |
|  |  |  |  | Not beneficial | | | | | | | | | | |  | |
|  |  |  |  | Felt tired | | | | | | | | | | |  | |
|  |  |  |  | Lack of support from family | | | | | | | | | | |  | |
|  |  |  |  | Social stigma | | | | | | | | | | |  | |
|  |  |  |  | Baby doesnot want to be in KMC position | | | | | | | | | | |  | |
|  |  |  |  | Healthy baby | | | | | | | | | | |  | |
|  |  |  |  | Others(Specify)……… …..…………..…… ……… | | | | | | | | | | | | |
| 1. **Perception of KMC wrap** | | | | | **Agree** | | | | | **Unsure** | | | | **Disagree** | | |
| - 1. It is comfortable to perform KMC with chosen wrap. | | | | |  | | | | |  | | | |  | | |
| - 1. The chosen wrap can hold the baby securely during KMC. | | | | |  | | | | |  | | | |  | | |
| 1. **Benefits of KMC** | | | | | | | | | | | | | | | | |
| - 1. What are the benefits of KMC?   ***(multiple response)*** | | | | | Facilitates breastfeeding | | | | | | | | |  | |  |
|  |  |  |  |  | Provides natural warmth to the baby | | | | | | | | |  | |  |
|  |  |  |  |  | Reduce infection for baby | | | | | | | | |  | |  |
|  |  |  |  |  | Promotes weight gain | | | | | | | | |  | |  |
|  |  |  |  |  | Easy to monitor condition of baby | | | | | | | | |  | |  |
|  |  |  |  |  | Facilitates mother and child bonding | | | | | | | | |  | |  |
|  |  |  |  |  | Others (specify)…………………………………… | | | | | | | | | | | |
| 1. **Spousal and Family acceptability** | | | | | | | | | | | | | | | | |
| - 1. Is your husband also providing KMC?   (doing KMC during your time for dinner, toilet, bathing, rest etc) | | | | | Yes | | | | | | No | | | NA | | |
| - 1. Is your family member also providing KMC?   (doing KMC during your time for dinner, toilet, bathing, rest etc) | | | | | Yes | | | | | | No | | | NA | | |
| **Section E: To be filled during phone survey (week 3)** | | | | | | | | | | | | | | | | |
| Hello!! Namaskar, My name is …………………..and I am calling from ……………………………………………. May I please speak to ………..? How are you? How is your baby? As you know, we are conducting a study on behalf of recently delivered mothers with stable preterm/LBW babies to explore adherence to KMC practice. As talked with you last week over the telephone. I am calling you to discuss about KMC practices at your home as continuation of the study. May I take your few minutes to ask few questions related to this? Thank you. | | | | | | | | | | | | | | | | |
| 1. **Background Information** | | | | | | | | | | | | | | | | |
| - 1. ID no. | | | | | - 1. Date of Follow up | | | | | | | | | | | |
| - 1. **Status of follow up** | | | | | | | | | | | | | | | | |
| 1.3.1 Able to Contact | | | | | 1.3.2 Not able to contact  (If not able to contact while contacting three times per day for three consecutive days) | | | | | | | | | | | |
| Reason for not being able to contact:……………………………………………….. | | | | | | | | | | | | | | | | |
| **Follow up questions** | | | | | | | | | | | | | | | | |
| 1. **Condition of mother and baby** | | | | | | | | | | | | | | | | |
| - 1. How are you? | Fine | | | | | | | |  | | | | **2.3** | | | |
|  | Not feeling well | | | | | | | |  | | | | | | | |
| - 1. What are the health problems?   (multiple response) | Fever | | | | | | | |  | | | | | | | |
|  | Pain in wound | | | | | | | |  | | | | | | | |
|  | Breast engorgement | | | | | | | |  | | | | | | | |
|  | Loos motion | | | | | | | |  | | | | | | | |
|  | Vomiting | | | | | | | |  | | | | | | | |
|  | Cough and congestion | | | | | | | |  | | | | | | | |
|  | Others specify………………………………………… | | | | | | | | | | | | | | | |
| - 1. How is your baby | Fine | | | | | | | |  | | | | 2.5 | | | |
|  | Not feeling well | | | | | | | |  | | | | | | | |
| - 1. What are the problems | Feeding problem | | | | | | | |  | | | | | | | |
|  | Sleeping problem | | | | | | | |  | | | | | | | |
|  | Fever | | | | | | | |  | | | | | | | |
|  | Loose motion | | | | | | | |  | | | | | | | |
|  | Vomiting | | | | | | | |  | | | | | | | |
|  | Cough and Congestion | | | | | | | |  | | | | | | | |
|  | Others specify………………………………………… | | | | | | | | | | | | | | | |
| - 1. Are you exclusively breastfeeding your baby? | | | Yes | | | | | No | | | | | | | | |
| 1. **KMC practice** | | | | | | | | | | | | | | | | |
| - 1. Are you continuing KMC at home? | | Yes | | | | | No  3.5 | | | | | | | | | |

| - 1. How many hours do you practice KMC per day (including both day and night) in this week.   . | **Dates** | **hours** |
| --- | --- | --- |
|  |  |  |
|  |  |  |
|  |  |  |
|  |  |  |
|  |  |  |
|  |  |  |
|  |  |  |

| - 1. Are you practicing KMC in day time while performing household chores? | | | | Yes | | | No | | | |
| --- | --- | --- | --- | --- | --- | --- | --- | --- | --- | --- |
| - 1. Are you practicing KMC at night? | | | | Yes | | | No | | | |
| - 1. Can you please provide the reason for discontinuation?   ***(multiple answers )*** | Physical pain following labor | | | | | | | | |  |
|  | Fear of harming baby through cord stump | | | | | | | | |  |
|  | Fear of baby falling | | | | | | | | |  |
|  | Discomfort | | | | | | | | |  |
|  | Lack confident | | | | | | | | |  |
|  | Lack of time | | | | | | | | |  |
|  | Not beneficial | | | | | | | | |  |
|  | Felt tired | | | | | | | | |  |
|  | Lack of support from family | | | | | | | | |  |
|  | Social stigma | | | | | | | | |  |
|  | Baby doesnot want to be in KMC position | | | | | | | | |  |
|  | Healthy baby | | | | | | | | |  |
|  | Others(Specify)……… …..…………..…… ……… | | | | | | | | | |
| 1. **Perception of KMC wrap** | | **Agree** | | | **Unsure** | | | **Disagree** | | |
| - 1. It is comfortable to perform KMC with chosen wrap. | |  | | |  | | |  | | |
| - 1. The chosen wrap can hold the baby securely during KMC. | |  | | |  | | |  | | |
| 1. **Benefits of KMC** | | | | | | | | | | |
| - 1. What are the benefits of KMC?   ***(multiple response)*** | | Facilitates breastfeeding | | | | | |  |  | |
|  |  | Provides natural warmth to the baby | | | | | |  |  | |
|  |  | Reduce infection for baby | | | | | |  |  | |
|  |  | Promotes weight gain | | | | | |  |  | |
|  |  | Easy to monitor condition of baby | | | | | |  |  | |
|  |  | Facilitates mother and child bonding | | | | | |  |  | |
|  |  | Others (specify)…………………………………… | | | | | | | | |
| 1. **Spousal and Family acceptability** | | | | | | | | | | |
| - 1. Is your husband also providing KMC?   (doing KMC during your time for dinner, toilet, bathing, rest etc) | | Yes | | | | No | | NA | | |
| - 1. Is your family member also providing KMC?   (doing KMC during your time for dinner, toilet, bathing, rest etc) | | Yes | | | | No | | NA | | |
| Thank you very much for participating in the study from the beginning. I would like to remind you for the PNC follow up visit at health facility next week. You will be provided with transportation cost of NRs 500. Thank you again. | | | | | | | | | | |
| **Section F: To be filled during PNC follow up at health facility (after week 4)** | | | | | | | | | | |
| 1. **Background Information** | | | | | | | | | | |
| - 1. ID no | | | - 1. Date of Follow up | | | | | | | |
| - 1. Weight of baby | | | - 1. Attended PNC YES NO | | | | | | | |

| **Follow up questions** | | | | | | | | | | | | | | | | | | | | | | |
| --- | --- | --- | --- | --- | --- | --- | --- | --- | --- | --- | --- | --- | --- | --- | --- | --- | --- | --- | --- | --- | --- | --- |
| 1. **Condition of mother and baby** | | | | | | | | | | | | | | | | | | | | | | |
| - 1. How are you? | Fine | | | | | | | | | | | |  | | | | | | **2.3** | | | |
|  | Not feeling well | | | | | | | | | | | |  | | | | | | | | | |
| - 1. What are the health problems?   (multiple response) | Fever | | | | | | | | | | | |  | | | | | | | | | |
|  | Pain in wound | | | | | | | | | | | |  | | | | | | | | | |
|  | Breast engorgement | | | | | | | | | | | |  | | | | | | | | | |
|  | Loose motion | | | | | | | | | | | |  | | | | | | | | | |
|  | Vomiting | | | | | | | | | | | |  | | | | | | | | | |
|  | Cough and congestion | | | | | | | | | | | |  | | | | | | | | | |
|  | Others specify………………………………………… | | | | | | | | | | | | | | | | | | | | | |
| - 1. How is your baby | Fine | | | | | | | | | | | |  | | | | | | 2.5 | | | |
|  | Not feeling well | | | | | | | | | | | |  | | | | | | | | | |
| - 1. What are the problems | Feeding problem | | | | | | | | | | | |  | | | | | | | | | |
|  | Sleeping problem | | | | | | | | | | | |  | | | | | | | | | |
|  | Fever | | | | | | | | | | | |  | | | | | | | | | |
|  | Loose motion | | | | | | | | | | | |  | | | | | | | | | |
|  | Vomiting | | | | | | | | | | | |  | | | | | | | | | |
|  | Cough and Congestion | | | | | | | | | | | |  | | | | | | | | | |
|  | Others specify………………………………………… | | | | | | | | | | | | | | | | | | | | | |
| - 1. Are you exclusively breastfeeding your baby? | | | Yes | | | | | | | | | No | | | | | | | | | | |
| 1. **KMC practice** | | | | | | | | | | | | | | | | | | | | | | |
| - 1. Are you continuing KMC at home? | | | | | | | | Yes | | | | | | | No 3.5 | | | | | | | |
| - 1. How many hours do you practice KMC per day (including both day and night) in this week | | **Dates** | | | | | | | | **hours** | | | | | | | | | | | | |
|  |  |  | | | | | | | |  | | | | | | | | | | | | |
|  |  |  | | | | | | | |  | | | | | | | | | | | | |
|  |  |  | | | | | | | |  | | | | | | | | | | | | |
|  |  |  | | | | | | | |  | | | | | | | | | | | | |
|  |  |  | | | | | | | |  | | | | | | | | | | | | |
|  |  |  | | | | | | | |  | | | | | | | | | | | | |
|  |  |  | | | | | | | |  | | | | | | | | | | | | |
| - 1. Are you practicing KMC at night? | | | | Yes | | | | | | | | | | | No | | | | | | | |
| - 1. Are you practicing KMC while performing household chores? | | | | Yes | | | | | | | | | | | No | | | | | | | |
| - 1. Can you please provide the reason for discontinuation?   ***(multiple answers )*** | | | | | Physical pain following labor | | | | | | | | | | | | | | | | |  |
|  |  |  |  |  | Fear of harming baby through cord stump | | | | | | | | | | | | | | | | |  |
|  |  |  |  |  | Fear of baby falling | | | | | | | | | | | | | | | | |  |
|  |  |  |  |  | Discomfort | | | | | | | | | | | | | | | | |  |
|  |  |  |  |  | Lack confident | | | | | | | | | | | | | | | | |  |
|  |  |  |  |  | Lack of time | | | | | | | | | | | | | | | | |  |
|  |  |  |  |  | Not beneficial | | | | | | | | | | | | | | | | |  |
|  |  |  |  |  | Felt tired | | | | | | | | | | | | | | | | |  |
|  |  |  |  |  | Lack of support from family | | | | | | | | | | | | | | | | |  |
|  |  |  |  |  | Social stigma | | | | | | | | | | | | | | | | |  |
|  |  |  |  |  | Baby doesnot want to be in KMC position | | | | | | | | | | | | | | | | |  |
|  |  |  |  |  | Healthy baby | | | | | | | | | | | | | | | | |  |
|  |  |  |  |  | Others(Specify)……… …..…………..…… ……… | | | | | | | | | | | | | | | | | |
| 1. **Perception of KMC wrap** | | | | | | | **Agree** | | | | | | | **Unsure** | | | | | | **Disagree** | | |
| - 1. It is comfortable to perform KMC with chosen wrap. | | | | | | |  | | | | | | |  | | | | | |  | | |
| - 1. The chosen wrap can hold the baby securely during KMC. | | | | | | |  | | | | | | |  | | | | | |  | | |
| 1. **Benefits of KMC** | | | | | | | | | | | | | | | | | | | | | | |
| - 1. What are the benefits of KMC?   ***(multiple response)*** | | | | | | | Facilitates breastfeeding | | | | | | | | | | | | |  |  | |
|  |  |  |  |  |  |  | Provides natural warmth to the baby | | | | | | | | | | | | |  |  | |
|  |  |  |  |  |  |  | Reduce infection for baby | | | | | | | | | | | | |  |  | |
|  |  |  |  |  |  |  | Promotes weight gain | | | | | | | | | | | | |  |  | |
|  |  |  |  |  |  |  | Easy to monitor condition of baby | | | | | | | | | | | | |  |  | |
|  |  |  |  |  |  |  | Facilitates mother and child bonding | | | | | | | | | | | | |  |  | |
|  |  |  |  |  |  |  | Others (specify)…………………………………… | | | | | | | | | | | | | | | |
| 1. **Family and Community acceptability** | | | | | | | | | | | | | | | | | | | | | | |
| - 1. Is your husband also providing KMC?   (doing KMC during your time for dinner, toilet, bathing, rest etc) | | | | | | Yes | | | | | No | | | | | | | NA | | | | |
| - 1. Is your family member also providing KMC?   (doing KMC during your time for dinner, toilet, bathing, rest etc) | | | | | | Yes | | | | | No | | | | | | | NA | | | | |
| - 1. What is the community attitude towards KMC? | | | | | | | | | Supportive | | | | | | | |  | | | | | |
|  |  |  |  |  |  |  |  |  | Surprise | | | | | | | |  | | | | | |
|  |  |  |  |  |  |  |  |  | To know more | | | | | | | |  | | | | | |
|  |  |  |  |  |  |  |  |  | Make fun of | | | | | | | |  | | | | | |
|  |  |  |  |  |  |  |  |  | No idea | | | | | | | |  | | | | | |
|  |  |  |  |  |  |  |  |  | Others**……………………….** | | | | | | | | | | | | | |
| 1. **Problems** | | | | | | | | | | | | | | | | | | | | | | |
| - 1. What were the practical problems while doing KMC? Please elaborate …………………………………………………………………………………………………………………………………………………………………………………… | | | | | | | | | | | | | | | | | | | | | | |
| 1. **Satisfaction and Recommendation** | | | | | | | | | | | | | | | | | | | | | | |
| - 1. Are you satisfied with the chosen wrap? | | | | | | | | | Yes  8.3 | | | | | | | No | | | | | | |
| - 1. Can you please provide the reason why you are not satisfied with the wrap?   …………………………………………………………………………………………………………………………………………………………………………………………………… | | | | | | | | | | | | | | | | | | | | | | |
| - 1. Do you recommend KMC to other mothers with preterm/LBW babies? | | | | | | | | | Yes | | | | | | | No | | | | | | |
| **Probe for reason** …………………………………………………………………………………………………………………………………………………………………………………………………………….. | | | | | | | | | | | | | | | | | | | | | | |
| - 1. Do you recommend the selected KMC wrap to other mothers with preterm/LBW babies? | | | | | | | | | Yes | | | | | | | No | | | | | | |
| **Probe for reason** …………………………………………………………………………………………………………………………………………………………………………………………………………….. | | | | | | | | | | | | | | | | | | | | | | |
| Thank participant for the continuing support from the beginning of the study and ask if she wants to share something important about the perception, usefulness and challenges or story/ verbatim of KMC practice and wrap that she might think has not been covered by the conversation till date.  **……………………………………………………………………………………………………………………………………………………………………………………………………………………………………………………………………………………………………** | | | | | | | | | | | | | | | | | | | | | | |

Thank you

1. Use HMIS ethnicity classification. [↑](#footnote-ref-1)
2. Completed years of schooling [↑](#footnote-ref-2)
