# Supplementary material for: Feasibility assessment of an ergonomic baby wrap for kangaroo mother care: A mixed methods study from Nepal
Source: PLoS One. 2018 Nov 15;13(11):e0207206. doi: 10.1371/journal.pone.0207206 (PMC6237334; doi:10.1371/journal.pone.0207206)
Supplement: S8 Fig — (DOCX) [file pone.0207206.s008.docx]

| **tflnd tyf jRrfnfO{ Gofgf] kfg]{ sk8fsf] (baby wrap) dfWod4f/f (KMC) ljlwnfO{ k\|f]T;fxg ug]{af/] cWoog** | | |
| --- | --- | --- |
| **dlxgf gk'uL hGd]sf÷hGdbfF sd tf}n ePsf lzz'x?sf] cfdfnfO{ :jf:Yo ;+:yfdf /xFbf, kmf]g ;j]{If0f / k\|;'tLkl5 kmnf]–ckdf hkfO{uf]sf] sd{rf/Ln] eg]{ kmf/fd** | | |
| **v08 s :jf:Yo ;+:yfaf6 l8:rfh{ x'g] ;dodf eg'{kg]{** | | |
| **!= k[i7e"dL** | | |
| !=! lhNnf | !=@ c:ktfn | |
| !=# lj/fdL egf{ g+= | !=$ kl/ro g+= | |
| !=% hft^!^ | !=^ z}lIfs cj:yf^@^ | |
| !=& gu/kflnsf÷uf= lj= ;= | !=* jf8{ g+ | |
| !=( pd]/ -k'/f ePsf] jif{_ | !=!) lhljt hGd]sf aRrfx?sf] ;+Vof | |
| !=!! k\|;'tL ePsf] ldlt | !=!@ ue{wf/0f xKtf | |
| **!=!#** k\|;'tLsf] lsl;d | ;fdfGo ?kn] ePsf]] | |
|  | kmf]/;]kn] tfg]/ ePsf] | |
|  | Eofs'dn] tfg]/ ePsf] | |
| !=!$ hGdbfFsf] tf}n -u\|fd_ | !=!% lnË | k'?if |
|  |  | dlxnf |
| !=!^ ;Dks{ gDa/ | | |

| **@=dfofsf] c+ufnf]] ;DalGw hfgsf/L** | | | | | | | | | | | | | | | |
| --- | --- | --- | --- | --- | --- | --- | --- | --- | --- | --- | --- | --- | --- | --- | --- |
| @=! dfofsf] c+ufnf]]sf] nflu egf{ ldlt | | @=@ dfofsf] c+ufnf]]sf] nflu egf{ ;do | | | | | | | | | | | | | |
| **@=# /]G8d ljefhgsf] >]0fL -Sequence of random allocation)** | | | | | | | | | | | | | | | |
| klxnf] k6s Gofgf] agfP/ a]/]sf] | ;'?jft ;do | | | | | | | | cGTo ;do | | | | | | |
| bf];\|f]] k6s Gofgf] agfP/ a]/]sf] | ;'?jft ;do | | | | | | | | cGTo ;do | | | | | | |
| @=$ afx\| 306fkl5 lzz'nfO{ Gofgf] kfg{ 5flgPsf] sk8f | | | | | | | | k/Dk/fut sk8f | | | | | | | |
|  |  |  |  |  |  |  |  | s]o/ Kn; | | | | | | | |
| @=% aRrf Gofgf] kfg]{ ljz]if sk8f /f]Hg'sf] sf/0f | | | | | | | | | | | | | | | |
| **sf/0fx?** | | | | | | | | | | **;xdt** | **clglZrt** | | | **c;xdt** | |
| ;'/lIft ePsf]n] | | | | | | | | | |  |  | | |  | |
| xnrn ug{ ;lsg] ePsf]n] | | | | | | | | | |  |  | | |  | |
| sd ylst x'g] ePsf]n] | | | | | | | | | |  |  | | |  | |
| k\|of]u ug{ ;lhnf] ePsf]n] | | | | | | | | | |  |  | | |  | |
| :tgkfg u/fpg ;lhnf] kfg]{ ePsf]n] | | | | | | | | | |  |  | | |  | |
| aRrfsf] ca:yf ;lhn} cg'udg ug{ ;lsg] ePsf]n] | | | | | | | | | |  |  | | |  | |
| n}+lus lje]b gePsf]n] -Gofgf] kfg{{ agfPsf] sk8fsf] gd'gf k'?if ;b:o tyf aRrfsf] afa'n] l7s dfg]sfn]]_ | | | | | | | | | |  |  | | |  | |
| cGo -pNn]v ug]{_ ================================================================================ | | | | | | | | | | | | | | | |
| @=^ aRrfnfO{ Gofgf] kfg{ 5flgPsf] sk8fsf] af/]df cfdfsf] dxTjk"0f{ wf/0ffx? -ToxL g} Gofgf] kfg]{ sk8f 5fGg'sf] sf/0f_  ======================================================================================================================================================================================================================================================================================================================================================================================================================================================================================== | | | | | | | | | | | | | | | |
| @=& aRrfnfO{ Gofgf] kfg{ 5flgPsf] sk8fsf] af/]df cfdfsf] dxTjk"0f{ cg'eax? -dfofsf] c+ufnf]]sf] cEof; ubf{ ef]u]sf ;d:ofx?_  **=========================================================================================================================================================================================================================================================================================================================================================================================================================================================================** | | | | | | | | | | | | | | | |
| @=* :jf:Yo ;+:yfdf a:bf dfofsf] c+ufnf]sf] cEof;sf] lsl;d -s[kof 306fdf n]Vg'xf];\_ | | | | | **lbgx?** | | **306f** | | | | | **;w}+ gu/]sf]**  *-k\|Zg g+ @=(df hfg]_* | | | |
|  |  |  |  |  | klxnf] lbg | |  | | | | |  | | | |
|  |  |  |  |  | bf];\|f] lbg | |  | | | | |  | | | |
|  |  |  |  |  | t];\|f] lbg | |  | | | | |  | | | |
|  |  |  |  |  | rf}yf] lbg | |  | | | | |  | | | |
|  |  |  |  |  | kfFrf} lbg | |  | | | | |  | | | |
|  |  |  |  |  | 5}6f} lbg | |  | | | | |  | | | |
|  |  |  |  |  | ;ftf} lbg | |  | | | | |  | | | |
|  |  |  |  |  | cf7f} lbg | |  | | | | |  | | | |
|  |  |  |  |  | gjf} lbg | |  | | | | |  | | | |
|  |  |  |  |  | bzf} lbg | |  | | | | |  | | | |
| @=( s[kof tkfO{n] lg/Gt/tf lbg g;Sg'sf] sf?0f atfOlbg'xf];\ <  **-ax'pQ/_** | | | | k\|;'tLkl5 zl// b'v]sf] sf/0fn] | | | | | | | | | | |  |
|  |  |  |  | gfeL Rofk]/ aRrfnfO{ ufx\|f] x'g] 8/n] | | | | | | | | | | |  |
|  |  |  |  | aRrf n8\g] 8/n] | | | | | | | | | | |  |
|  |  |  |  | c;lhnf] eP/ | | | | | | | | | | |  |
|  |  |  |  | ljZjf; gnfu]sf]n] | | | | | | | | | | |  |
|  |  |  |  | ;dosf] cefjn] | | | | | | | | | | |  |
|  |  |  |  | kmfObf gePsf]n] | | | | | | | | | | |  |
|  |  |  |  | ylst ePsf]n] | | | | | | | | | | |  |
|  |  |  |  | kl/jf/sf] ;xof]u gePsf]n] | | | | | | | | | | |  |
|  |  |  |  | ;fdflhs cGwljZjf;sf] sf/0fn] | | | | | | | | | | |  |
|  |  |  |  | aRrf dfofsf] c+ufnf]]df a:g gdfg]sf]n] | | | | | | | | | | |  |
|  |  |  |  | aRrf :j:y ePsf]n] | | | | | | | | | | |  |
|  |  |  |  | cGo -pNn]v ug]{_ ================================================== | | | | | | | | | | | |
| @=!) c:ktfnaf6 l8:rfh{ x'Fbf aRrfsf] tf}n -u\|fd_ | | | | | | | | | | | | | | | |
| @=!! :jf:YosdL{x?sf] cjnf]sg -aRrfnfO{ Gofgf] kfg]{ g5flgPsf] tyf 5flgPsf] sk8f k\|of]u / cEof; ;DaGwdf dxTjk'0f{ cjnf]sg_  ======================================================================================================================================================================================================================================================================================================================================================================================================================================================================================================================= | | | | | | | | | | | | | | | |
| **v08 vM kmf]g ;e]{If0fsf] a]nf eg'{kg]{ -l8:rfh{ ePsf] @$ 306fkl5 / $* 306f leq_** | | | | | | | | | | | | | | | |
| x]nf], gd:sf/, d]/f] gfd================== xf] / d ================= af6 af]ln/x]sf] 5' . s[kof, s] d ================== ;Fu s'/f ug{ ;S5' . tkfO{ / tkfO{sf] aRrfnfO{ s:tf] 5 < tkfO{nfO{ yfxf 5 xfdL xfn;fn} k\|;'tL u/fPsf cfdfx?nfO{ dfofsf] c+ufnf]]sf] cEof;sf] lg/Gt/tfsf] af/]df cg';Gwfg ul//x]sf 5f} . tkfO{n] ======================= c:ktfndf k\|;'tL u/fpFbf o; cWoogdf ;xefuL x'g OR5's ePsf] hfgsf/L u/fpg' ePsf] lyof] . o; cWoognfO{ lg/Gt/tf lbb} tkfO{sf] 3/df dfofsf] c+ufnf]]sf] cEof; ;DalGw 5nkmn ug{ tkfO{nfO{ kmf]g u/]sf] 5' . s] d tkfO{sf] s]xL ;do o; ljifodf 5nkmn ug{ lng ;S5' < wGojfb . | | | | | | | | | | | | | | | |
| **!= k[i7e"dL** | | | | | | | | | | | | | | | |
| !=! kl/ro g+= | | | | | | !=@ kmnf]–cksf] ldlt | | | | | | | | | |
| **!=# kmnf]–cksf] cj:yf** | | | | | | | | | | | | | | | |
| !=#=! ;Dks{ ug{ ;lsPsf] | | | | | | !=#=@ ;Dks{ ug{ g;lsPsf] | | | | | | | | | |
| ;Dks{ ug{ g;Sg'sf] sf/0f ========================================================================== | | | | | | | | | | | | | | | |
| **kmnf]–ck ;DalGw k\|Zgx?** | | | | | | | | | | | | | | | |
| **@= cfdf / aRrfsf] cj:yf** | | | | | | | | | | | | | | | |
| @=! tkfO{nfO{{ s:tf] 5 < | | | l7s 5 | | |  | | | | | | | @=# | | |
|  |  |  | ;Grf] 5}g | | |  | | | | | | | | | |

| @=@ tkfO{sf :jf:Yo ;d:ofx? s]–s] x'g\ <  **-ax'pQ/ cfpg ;S5_** | Hj/f] |  | |
| --- | --- | --- | --- |
|  | 3fpsf] b'vfO |  | |
|  | b'w uflgPsf] |  | |
|  | kftnf] lb;f |  | |
|  | jfGtf |  | |
|  | ?3f vf]sL |  | |
|  | cGo -pNn]v ug]{_ ======================================= | | |
| @=# tkfO{sf] aRrfnfO{ s:tf] 5 < | l7s 5 |  | @=% |
|  | ;Grf] 5}g |  | |
| @=$ ;d:ofx? s]–s] 5g\ < | v'jfpg] ;d:of |  | |
|  | ;'tfpg] ;d:of |  | |
|  | Hj/f] cfPsf] 5 |  | |
|  | kftnf] lb;f nfu]sf] 5 |  | |
|  | jfGtf eO/x]sf] 5 |  | |
|  | ?3f vf]sL nfu]sf] 5 |  | |
|  | cGo -pNn]v ug]{_ ======================= | | |
| @=% tkfO{n] cfkm\gf] aRrfnfO{ :tgkfg dfq u/fO/xg' ePsf] 5 < | 5 | 5}g | |

| **#= dfofsf] c+ufnf]]sf] cEof;** | | | | | | | | |
| --- | --- | --- | --- | --- | --- | --- | --- | --- |
| #=! s] tkfO{ 3/df dfofsf] c+ufnf]]nfO{ lg/Gt/tf lbO/xg' ePsf] 5 < | 5 | | 5}g  3.5 | | | | | |
| #=@ ljut @$ 306fdf tkfO{n] slt 306f dfofsf] c+ufnf]] df /fVg'eof]] < |  | | | | | | | |
| #=# s] tkfO{ 3/sf] sfdsfh ubf{ klg dfofsf] c+ufnf]] cEof; ug'{x'G5 < | u5'{ | | | ulb{g | | | | |
| #=$ s] tkfO{ /flt dfofsf] c+ufnf]]sf] cEof; ug'{x'G5 < | u5'{ | | | ulb{g | | | | |
| #=% tkfO{n] dfofsf] c+ufnf]nfO{ lg/Gt/tf glbg'sf] sf/0f s] xf] < **-ax'pQ/_** | k\|;'tLkl5 z/L/ b'v]sf] sf/0fn] | | | | |  | | |
|  | gfeL Rofk]/ aRrfnfO{ ufx\|f] x'g] 8/n] | | | | |  | | |
|  | aRrf n8\g] 8/n] | | | | |  | | |
|  | c;lhnf] eP/ | | | | |  | | |
|  | ljZjf; gnfu]sf]n] | | | | |  | | |
|  | ;dosf] cefjn] | | | | |  | | |
|  | kmfObf gePsf]n] | | | | |  | | |
|  | ylst ePsf]n] | | | | |  | | |
|  | kl/jf/sf] ;xof]u gePsf]n] | | | | |  | | |
|  | ;fdflhs cGwljZjf;sf] sf/0fn] | | | | |  | | |
|  | aRrf dfofsf] c+ufnf]]df a:g gdfg]sf]n] | | | | |  | | |
|  | aRrf :j:y ePsf]n] | | | | |  | | |
|  | cGo -pNn]v ug]{_ ======================= | | | | | | | |
| **$=** dfofsf] c+ufnf]]df Gofgf] kfg]{ sk8f ;DalGw wf/0ff | | | | **;xdt** | **clglZrt** | | **c;xdt** | |
| $=! /f]lhPsf] Gofgf] kfg]{ sk8fn] dfofsf] c+ufnf]]df k\|:t't ug{ ;lhnf] tyf cf/fbfoL x'g] . | | | |  |  | |  | |
| $=@ /f]lhPsf] Gofgf] kfg]{ sk8fn] aRrfnfO{ dfofsf] c+ufnf]]df ;'/lIft ;fy af]Sg ;lsg] . | | | |  |  | |  | |
| **%= dfofsf] c+ufnf]]sf kmfObfx?** | | | | | | | | |
| %=! dfofsf] c+ufnf]]sf kmfObfx? s]–s] x'g\ <  **-ax'pQ/_** | | :tgkfg u/fpg ;lhnf] x'G5 | | | | | |  |
|  |  | aRrfn] k\|fs[lts Gofgf]kg kfpF5 | | | | | |  |
|  |  | aRrfsf] ;+s\|d0f 36fpF5 | | | | | |  |
|  |  | tf}n a9\g d2t u5{ | | | | | |  |
|  |  | aRrfsf] cj:yf cg'udg ug{ ;lhnf] | | | | | |  |
|  |  | cfdf / aRrfsf] ;DaGw k\|uf9 gfpF5 | | | | | |  |
|  |  | cGo -pNn]v ug]{_ ======================= | | | | | | |

| **^= >Ldfg / kl/jf/sf] :jLsfo{tf** | | | | | | |
| --- | --- | --- | --- | --- | --- | --- |
| ^=! s] tkfO{sf] >Ldfgn] klg dfofsf] c+ufnf]] lbg'x'G5 <  -tkfO{ vfgf vfFbf, 6\jfOn]6 hfFbf, g'xfpFbf, cf/fd ubf{ cflb_ | | lbg'x'G5 | lbg'x'b}g | | | nfu" x'b}g |
| ^=@ s] tkfO{sf kl/jf/sf ;b:ox?n] klg dfofsf] c+ufnf]] lbg'x'G5 <  -tkfO{ vfgf vfFbf, 6\jfOn]6 hfFbf, g'xfpFbf, cf/fd ubf{ cflb_ | | lbg'x'G5 | lbg'x'b}g | | | nfu" x'b}g |
| **v08 uM kmf]g ;e]{If0fsf] a]nf eg'{kg]{ -klxnf] xKtf_** | | | | | | |
| x]nf], gd:sf/, d]/f] gfd================== xf] / d ================= af6 af]ln/x]sf] 5' . s[kof, s] d ================== ;Fu s'/f ug{ ;S5' . tkfO{ / tkfO{sf] aRrfnfO{ s:tf] 5 < tkfO{nfO{ yfxf 5 xfdL xfn;fn} k\|;'tL u/fPsf cfdfx?nfO{ dfofsf] c+ufnf]]sf] cEof;sf] lg/Gt/tfsf] af/]df cg';Gwfg ul//x]sf 5f} . tkfO{n] ======================= c:ktfndf k\|;'tL u/fpFbf o; cWoogdf ;xefuL x'g OR5's ePsf] hfgsf/L u/fpg' ePsf] lyof] . o; cWoognfO{ lg/Gt/tf lbb} tkfO{sf] 3/df dfofsf] c+ufnf]]sf] cEof; ;DalGw 5nkmn ug{ tkfO{nfO{ kmf]g u/]sf] 5' . s] d tkfO{sf] s]xL ;do o; ljifodf 5nkmn ug{ lng ;S5' < wGojfb . | | | | | | |
| **!= k[i7e"dL** | | | | | | |
| !=! kl/ro g+= | | !=@ kmnf]–cksf] ldlt | | | | |
| **!=# kmnf]–cksf] cj:yf** | | | | | | |
| !=#=! ;Dks{ ug{ ;lsPsf] | | !=#=@ ;Dks{ ug{ g;lsPsf]  -;Dks{ ug{ g;s]sf] eP # lbg;Dd k\|To]s lbg # k6s;Dd ;Dks{ ug{ k\|of; ug]{_ | | | | |
| ;Dks{ ug{ g;Sg'sf] sf/0f ========================================================================== | | | | | | |
| **kmnf]–ck ;DalGw k\|Zgx?** | | | | | | |
| **@= cfdf / aRrfsf] cj:yf** | | | | | | |
| @=! tkfO{nfO{{ s:tf] 5 < | l7s 5 |  | | **@=#** | | |
|  | ;Grf] 5}g |  | | | | |
| @=@ tkfO{sf :jf:Yo ;d:ofx? s]–s] x'g\ <  **-ax'pQ/_** | Hj/f] |  | | | | |
|  | 3fpsf] b'vfO |  | | | | |
|  | b'w uflgPsf] |  | | | | |
|  | kftnf] lb;f |  | | | | |
|  | jfGtf |  | | | | |
|  | ?3f vf]sL |  | | | | |
|  | cGo -pNn]v ug]{_ ==================================================== | | | | | |
| @=# tkfO{sf] aRrfnfO{ s:tf] 5 < | l7s 5 |  | | | @=% | |
|  | ;Grf] 5}g |  | | | | |

| @=$ ;d:ofx? s]–s] 5g\ < | v'jfpg] ;d:of |  |
| --- | --- | --- |
|  | ;'tfpg] ;d:of |  |
|  | Hj/f] cfPsf] 5 |  |
|  | kftnf] lb;f nfu]sf] 5 |  |
|  | jfGtf eO/x]sf] 5 |  |
|  | ?3f vf]sL nfu]sf] 5 |  |

|  | cGo -pNn]v ug]{_ ======================= | | |
| --- | --- | --- | --- |
| @=% tkfO{n] cfkm\gf] aRrfnfO{ :tgkfg dfq u/fO/xg' ePsf] 5 < | | 5 | 5}g |

| **#= dfofsf] c+ufnf]]sf] cEof;** | | | | | |
| --- | --- | --- | --- | --- | --- |
| #=! s] tkfO{ 3/df dfofsf] c+ufnf]]nfO{ lg/Gt/tf lbO/xg' ePsf] 5 **<** | 5 | | 5}g | | |
| #=@ gjhft lzz'nfO{ lbgsf] slt 306f **dfofsf] c+ufnf]] df /fVg'eof]** < -lbg / /ft u/]/_ | ldlt | | 306f | | |
|  |  | |  | | |
|  |  | |  | | |
|  |  | |  | | |
| **v08 3M kmf]g ;e]{If0fsf] a]nf eg'{kg]{ -bf];\|f] xKtf_** | | | | | |
| x]nf], gd:sf/, d]/f] gfd================== xf] / d ================= af6 af]ln/x]sf] 5' . s[kof, s] d ================== ;Fu s'/f ug{ ;S5' . tkfO{ / tkfO{sf] aRrfnfO{ s:tf] 5 < tkfO{nfO{ yfxf 5 xfdL xfn;fn} k\|;'tL u/fPsf cfdfx?nfO{ dfofsf] c+ufnf]]sf] cEof;sf] lg/Gt/tfsf] af/]df cg';Gwfg ul//x]sf 5f} . ut xKtf 6]lnkmf]gdf s'/f u/] h:t} cWoognfO{ lg/Gt/tf lbg tkfO{sf] 3/df dfofsf] c+ufnf]]sf] cEof; ;DalGw 5nkmn ug{ tkfO{nfO{ kmf]g u/]sf] 5' . s] d o; ljifodf k\|Zg ug{ tkfO{sf] s]xL ;do lng ;S5' < wGojfb . | | | | | |
| **!= k[i7e"dL** | | | | | |
| !=! kl/ro g+= | !=@ kmnf]–cksf] ldlt | | | | |
| **!=# kmnf]–cksf] cj:yf** | | | | | |
| !=#=! ;Dks{ ug{ ;lsPsf] | !=#=@ ;Dks{ ug{ g;lsPsf]  -;Dks{ ug{ g;s]sf] eP # lbg;Dd k\|To]s lbg # k6s;Dd ;Dks{ ug{ k\|of; ug]{_ | | | | |
| ;Dks{ ug{ g;Sg'sf] sf/0f ========================================================================== | | | | | |
| **kmnf]–ck ;DalGw k\|Zgx?** | | | | | |
| **@= cfdf / aRrfsf] cj:yf** | | | | | |
| @=! tkfO{nfO{{ s:tf] 5 < | | l7s 5 | |  | @=# |
|  |  | ;Grf] 5}g | |  | |
| @=@ tkfO{sf :jf:Yo ;d:ofx? s]–s] x'g\ <  **-ax'pQ/_** | | Hj/f] | |  | |
|  |  | 3fpsf] b'vfO | |  | |
|  |  | b'w uflgPsf] | |  | |
|  |  | kftnf] lb;f | |  | |
|  |  | jfGtf | |  | |
|  |  | ?3f vf]sL | |  | |
|  |  | cGo -pNn]v ug]{_ ========================================== | | | |

| @=# tkfO{sf] aRrfnfO{ s:tf] 5 < | l7s 5 |  | **@=%** |
| --- | --- | --- | --- |
|  | ;Grf] 5}g |  | |
| @=$ ;d:ofx? s]–s] 5g\ < | v'jfpg] ;d:of |  | |
|  | ;'tfpg] ;d:of |  | |
|  | Hj/f] cfPsf] 5 |  | |
|  | kftnf] lb;f nfu]sf] 5 |  | |
|  | jfGtf eO/x]sf] 5 |  | |
|  | ?3f vf]sL nfu]sf] 5 |  | |
|  | cGo -pNn]v ug]{_ ======================= | | |
| @=% tkfO{n] cfkm\gf] aRrfnfO{ :tgkfg dfq u/fO/xg' ePsf] 5 < | 5 | 5}g | |
| **#= dfofsf] c+ufnf]]sf] cEof;** | | | |
| #=! s] tkfO{ 3/df dfofsf] c+ufnf]]nfO{ lg/Gt/tf lbO/xg' ePsf] 5 < | 5 | 5}g  #=% | |
| #=@ uPsf] xKtfdf, gjhft lzz'nfO{ lbgsf] slt 306f **dfofsf] c+ufnf]] df /fVg'eof]** < -lbg / /ft u/]/_ | ldlt | 306f | |
|  |  |  | |
|  |  |  | |
|  |  |  | |
|  |  |  | |
|  |  |  | |
|  |  |  | |
|  |  |  | |
|  |  |  | |
| #=# s] tkfO{ 3/sf] sfdsfh ubf{ klg dfofsf] c+ufnf]sf]] cEof; ug'{x'G5 < | u5'{ | ulb{g | |
| #=$ s] tkfO{ /flt dfofsf] c+ufnf]] cEof; ug'{x'G5 < | u5'{ | ulb{g | |

| #=% tkfO{ lg/Gt/tf glbg'sf] sf/0f s] xf] <  **-ax'pQ/_** | k\|;'tLkl5 z/L/ b'v]sf] sf/0fn] |  |
| --- | --- | --- |
|  | gfeL Rofk]/ aRrfnfO{ ufx\|f] x'g] 8/n] |  |
|  | aRrf n8\g] 8/n] |  |
|  | c;lhnf] eP/ |  |
|  | ljZjf; gnfu]sf]n] |  |
|  | ;dosf] cefjn] |  |
|  | kmfObf gePsf]n] |  |
|  | ylst ePsf]n] |  |
|  | kl/jf/sf] ;xof]u gePsf]n] |  |
|  | ;fdflhs cGwljZjf;sf] sf/0fn] |  |
|  | aRrf dfofsf] c+ufnf]]df a:g gdfg]sf]n] |  |
|  | aRrf :j:y ePsf]n] |  |
|  | cGo -pNn]v ug]{_ ======================= | |

| **$=** dfofsf] c+ufnf]]df Gofgf] kfg]{ sk8f ;DalGw wf/0ff | **;xdt** | **clglZrt** | **c;xdt** |
| --- | --- | --- | --- |
| $=! /f]lhPsf] Gofgf] kfg]{ sk8fn] dfofsf] c+ufnf]]df /fVg ;lhnf] tyf cf/fdbfoL x'g] . |  |  |  |
| $=@ /f]lhPsf] Gofgf] kfg]{ sk8fn] aRrfnfO{ dfofsf] c+ufnf]]df ;'/lIft ;fy af]Sg ;lsg] . |  |  |  |

| %= dfofsf] c+ufnf]]sf kmfObfx? | | |
| --- | --- | --- |
| %=! dfofsf] c+ufnf]]sf kmfObfx? s]–s] x'g\ < **-ax'pQ/_** | :tgkfg u/fpg ;lhnf] x'G5 |  |
|  | aRrfn] k\|fs[lts Gofgf]kg kfpF5 |  |
|  | aRrfsf] ;+s\|d0f 36fpF5 |  |
|  | tf}n a9\g d2t u5{ |  |
|  | aRrfsf] cj:yf cg'udg ug{ ;lhnf] x'G5 |  |
|  | cfdf / aRrfsf] ;DaGw k\|uf9 agfpF5 |  |
|  | cGo -pNn]v ug]{_ ======================= | |

| **^= >Ldfg / kl/jf/sf] :jLsfo{tf** | | | |
| --- | --- | --- | --- |
| ^=! s] tkfO{sf] >Ldfgn] klg dfofsf] c+ufnf]] lbg'x'G5 <  -tkfO{ vfgf vfFbf, 6\jfOn]6 hfFbf, g'xfpFbf, cf/fd ubf{ cflb_ | lbg'x'G5 | lbg'x'b}g | nfu" x'b}g |
| ^=@ s] tkfO{sf kl/jf/sf ;b:ox?n] klg dfofsf] c+ufnf]] lbg'x'G5 <  -tkfO{ vfgf vfFbf, 6\jfOn]6 hfFbf, g'xfpFbf, cf/fd ubf{ cflb_ | lbg'x'G5 | lbg'x'b}g | nfu" x'b}g |

| **v08 ª M kmf]g ;e]{If0fsf] a]nf eg'{kg]{ -t];\|f] xKtf_** | |
| --- | --- |
| x]nf], gd:sf/, d]/f] gfd================== xf] / d ================= af6 af]ln/x]sf] 5' . s[kof, s] d ================== ;Fu s'/f ug{ ;S5' . tkfO{ / tkfO{sf] aRrfnfO{ s:tf] 5 < tkfO{nfO{ yfxf 5 xfdL xfn;fn} k\|;'tL u/fPsf cfdfx?nfO{ dfofsf] c+ufnf]]sf] cEof;sf] lg/Gt/tfsf] af/]df cg';Gwfg ul//x]sf 5f} . ut xKtf 6]lnkmf]gdf s'/f u/] h:t} cWoognfO{ lg/Gt/tf lbg tkfO{sf] 3/df dfofsf] c+ufnf]]sf] cEof; ;DalGw 5nkmn ug{ tkfO{nfO{ kmf]g u/]sf] 5' . s] d o; ljifodf k\|Zg ug{ tkfO{sf] s]xL ;do lng ;S5' < wGojfb . | |
| **!= k[i7e"dL** | |
| !=! kl/ro g+= | !=@ kmnf]–cksf] ldlt |
| !=# kmnf]–cksf] cj:yf | |
| !=#=! ;Dks{ ug{ ;lsPsf] | !=#=@ ;Dks{ ug{ g;lsPsf]  -;Dks{ ug{ g;s]sf] eP # lbg;Dd k\|To]s lbg # k6s;Dd ;Dks{ ug{ k\|of; ug]{_ |
| ;Dks{ ug{ g;Sg'sf] sf/0f ========================================================================== | |

| **kmnf]–ck ;DalGw k\|Zgx?** | | | | | | |
| --- | --- | --- | --- | --- | --- | --- |
| **@= cfdf / aRrfsf] cj:yf** | | | | | | |
| @=! tkfO{nfO{{ s:tf] 5 < | l7s 5 | |  | | | @=# |
|  | ;Grf] 5}g | |  | | | |
| @=@ tkfO{sf :jf:Yo ;d:of s]–s] x'g\ < **-ax'pQ/ _** | Hj/f] | |  | | | |
|  | 3fpsf] b'vfO | |  | | | |
|  | b'w uflgPsf] | |  | | | |
|  | kftnf] lb;f | |  | | | |
|  | jfGtf | |  | | | |
|  | ?3f vf]sL | |  | | | |
|  | cGo -pNn]v ug]{_ ============================================= | | | | | |
| @=# tkfO{sf] aRrfnfO{ s:tf] 5 < | l7s 5 | |  | | **@=%** | |
|  | ;Grf] 5}g | |  | | | |
| **@=$ ;d:ofx? s]–s] 5g\ <** | v'jfpg] ;d:of | |  | | | |
|  | ;'tfpg] ;d:of | |  | | | |
|  | Hj/f] cfPsf] 5 | |  | | | |
|  | kftnf] lb;f nfu]sf] 5 | |  | | | |
|  | jfGtf eO/x]sf] 5 | |  | | | |
|  | ?3f vf]sL nfu]sf] 5 | |  | | | |
|  | cGo -pNn]v ug]{_ ======================= | | | | | |
| @=% tkfO{n] cfkm\gf] aRrfnfO{ :tgkfg dfq u/fO/xg' ePsf] 5< | 5 | | | 5}g | | |
| **#= dfofsf] c+ufnf]]sf] cEof;** | | | | | | |
| #=! s] tkfO{ 3/df dfofsf] c+ufnf]]nfO{ lg/Gt/tf lbO/xg' ePsf] 5 < | | 5 | | 5}g  #=% | | |
| #=@ uPsf] xKtfdf, gjhft lzz'nfO{ lbgsf] slt 306f **dfofsf] c+ufnf]] df /fVg'eof]** < -lbg / /ft u/]/_ | | ldlt | | 306f | | |
|  |  |  | |  | | |
|  |  |  | |  | | |
|  |  |  | |  | | |
|  |  |  | |  | | |
|  |  |  | |  | | |
|  |  |  | |  | | |
|  |  |  | |  | | |
| #=# s] tkfO{ 3/sf] sfdsfh ubf{ klg dfofsf] c+ufnf]] cEof; ug'{x'G5 < | | u5'{ | | ulb{g | | |
| #=$ s] tkfO{ /flt dfofsf] c+ufnf]] cEof; ug'{x'G5 < | | u5'{ | | ulb{g | | |

| #=% tkfO{n]  **lg/Gt/tf glbg'sf] sf/0f s] xf] <**  **-ax'pQ/_** | | k\|;'tLkl5 z/L/ b'v]sf] sf/0fn] | | | | |  |
| --- | --- | --- | --- | --- | --- | --- | --- |
|  |  | gfeL Rofk]/ aRrfnfO{ ufx\|f] x'g] 8/n] | | | | |  |
|  |  | aRrf n8\g] 8/n] | | | | |  |
|  |  | c;lhnf] eP/ | | | | |  |
|  |  | ljZjf; gnfu]sf]n] | | | | |  |
|  |  | ;dosf] cefjn] | | | | |  |
|  |  | kmfObf gePsf]n] | | | | |  |
|  |  | ylst ePsf]n] | | | | |  |
|  |  | kl/jf/sf] ;xof]u gePsf]n] | | | | |  |
|  |  | ;fdflhs cGwljZjf;sf] sf/0fn] | | | | |  |
|  |  | aRrf dfofsf] c+ufnf]]df a:g gdfg]sf]n] | | | | |  |
|  |  | aRrf :j:y ePsf]n] | | | | |  |
|  |  | cGo -pNn]v ug]{_ ======================= | | | | | |
| **$=** dfofsf] c+ufnf]]df Gofgf] kfg]{ sk8f ;DalGw wf/0ff | | **;xdt** | **clglZrt** | **c;xdt** | | | |
| $=! /f]lhPsf] Gofgf] kfg]{ sk8fn] dfofsf] c+ufnf]]df /fVg ;lhnf tyf cf/fdbfoL x'g] . | |  |  |  | | | |
| $=@ /f]lhPsf] Gofgf] kfg]{ sk8fn] aRrfnfO{ dfofsf] c+ufnf]]df ;'/lIft ;fy af]Sg ;lsg] . | |  |  |  | | | |
| %= dfofsf] c+ufnf]]sf kmfObfx? | | | | | | | |
| %=! dfofsf] c+ufnf]]sf kmfObfx? s]–s] x'g\ <  **-ax'pQ/_** | | :tgkfg u/fpg ;lhnf] x'G5 | | |  | | |
|  |  | aRrfn] k\|fs[lts Gofgf]kg kfpF5 | | |  | | |
|  |  | aRrfsf] ;+s\|d0f 36fpF5 | | |  | | |
|  |  | tf}n a9\g d2t u5{ | | |  | | |
|  |  | aRrfsf] cj:yf cg'udg ug{ ;lhnf] x'G5 | | |  | | |
|  |  | cfdf / aRrfsf] ;DaGw k\|uf9 agfpF5 | | |  | | |
|  | | cGo -pNn]v ug]{_==================== | | |  | | |
| ^=! s] tkfO{sf] >Ldfgn] klg dfofsf] c+ufnf]] lbg'x'G5 <  -tkfO{ vfgf vfFbf, 6\jfOn]6 hfFbf, g'xfpFbf, cf/fd ubf{ cflb_ | | lbg'x'G5 | lbg'x'b}g | | | nfu" x'b}g | |
| ^=@ s] tkfO{sf kl/jf/sf ;b:ox?n] klg dfofsf] c+ufnf]] lbg'x'G5 <  -tkfO{ vfgf vfFbf, 6\jfOn]6 hfFbf, g'xfpFbf, cf/fd ubf{ cflb_ | | lbg'x'G5 | lbg'x'b}g | | | nfu" x'b}g | |
| **o; cWoogdf ;'?blv clxn];Dd ;xefuL eO{lbg' ePsf]df wGojfb . d oxfFnfO{ ;'Ts]/L kl5sf] kl/If0fsf] nflu csf]{ xKtf :jf:Yo ;+:yf cfpgsf] nflu ;Demfpg rxfG5' . oxfFnfO{ To; a]nf oftfoft vr{ eg]/ ? %)) lbOG5 . wGojfb .** | | | | | | | |
| **v08 rM :jf:Yo ;+:yfdf ;'Ts]/L kl5sf] kl/If0fsf] a]nf eg]{ -$ xKtf kl5_** | | | | | | | |
| **!= k[i7e"dL** | | | | | | | |
| !=! kl/ro g+= | !=@ kmnf]–cksf] ldlt | | | | | | |
| !=# aRrfsf] tf}n | !=$ ;'Ts]/L kl5sf] kl/If0f u/fPsf]  xf] xf]Og | | | | | | |
| kmnf]–ck ;DalGw k\|Zgx? | | | | | | | |
| **@= cfdf / aRrfsf] cj:yf** | | | | | | | |

| @=! tkfO{nfO{{ s:tf] 5 < | l7s 5 | **@=#** |
| --- | --- | --- |
|  | ;Grf] 5}g |  |
| @=@ tkfO{sf :jf:Yo ;d:ofx? s]–s] x'g\ <  **-ax'pQ/_** | Hj/f] |  |
|  | 3fpsf] b'vfO |  |
|  | b'w uflgPsf] |  |
|  | kftnf] lb;f |  |
|  | jfGtf |  |
|  | ?3f vf]sL |  |
|  | cGo -pNn]v ug]{_ =============================================== | |
| @=# tkfO{sf] aRrfnfO{ s:tf] 5 < | l7s 5 | **@=%** |
|  | ;Grf] 5}g |  |
| @=$ ;d:ofx? s]–s] 5g\ < | v'jfpg] ;d:of |  |
|  | ;'tfpg] ;d:of |  |
|  | Hj/f] cfPsf] 5 |  |
|  | kftnf] lb;f nfu]sf] 5 |  |
|  | jfGtf eO/x]sf] 5 |  |
|  | ?3f vf]sL nfu]sf] 5 |  |
|  | cGo -pNn]v ug]{_ ======================= | |

| @=% tkfO{n] cfkm\gf] aRrfnfO{ :tgkfg dfq u/fO/xg' ePsf] 5 < | 5 | 5}g |
| --- | --- | --- |
| **#= dfofsf] c+ufnf]]sf] cEof;** | | |
| #=! s] tkfO{ 3/df dfofsf] c+ufnf]]nfO{ lg/Gt/tf lbO/xg' ePsf] 5 < | 5 | 5}g  #=% |
| #=@ uPsf] xKtfdf, gjhft lzz'nfO{ lbgsf] slt 306f dfofsf] c+ufnf]] df /fVg'eof] <  -lbg / /ft u/]/_ | ldlt | 306f |
|  |  |  |
|  |  |  |
|  |  |  |
|  |  |  |
|  |  |  |
|  |  |  |
|  |  |  |
|  |  |  |
| #=# s] tkfO{ 3/sf] sfdsfh ubf{ klg dfofsf] c+ufnf]] cEof; ug'{x'G5 < | u5'{ | ulb{g |
| #=$ s] tkfO{ /flt dfofsf] c+ufnf]] cEof; ug'{x'G5 < | u5'{ | ulb{g |

| #=% tkfO{n] lg/Gt/tf glbg'sf] sf/0f s] xf] <  **-ax'pQ/_** | k\|;'tLkl5 z/L/ b'v]sf] sf/0fn] | | |  |
| --- | --- | --- | --- | --- |
|  | gfeL Rofk]/ aRrfnfO{ ufx\|f] x'g] 8/n] | | |  |
|  | aRrf n8\g] 8/n] | | |  |
|  | c;lhnf] eP/ | | |  |
|  | ljZjf; gnfu]sf]n] | | |  |
|  | ;dosf] cefjn] | | |  |
|  | kmfObf gePsf]n] | | |  |
|  | ylst ePsf]n] | | |  |
|  | kl/jf/sf] ;xof]u gePsf]n] | | |  |
|  | ;fdflhs cGwljZjf;sf] sf/0fn] | | |  |
|  | aRrf dfofsf] c+ufnf]]df a:g gdfg]sf]n] | | |  |
|  | aRrf :j:y ePsf]n] | | |  |
|  | cGo -pNn]v ug]{_ ======================= | | | |
| **$=** dfofsf] c+ufnf]]df Gofgf] kfg]{ ;DalGw wf/0ff | **;xdt** | **clglZrt** | **c;xdt** | |
| $=! /f]lhPsf] Gofgf] kfg]{ sk8fn] dfofsf] c+ufnf]]df /fVg ;lhnf] tyf cf/fdbfoL x'g] . |  |  |  | |
| $=@ /f]lhPsf] Gofgf] kfg]{ sk8fn] aRrfnfO{ dfofsf] c+ufnf]]df ;'/lIft ;fy af]Sg ;lsg] . |  |  |  | |
| %= **dfofsf] c+ufnf]]sf kmfObfx?** | | | | |

| %=! dfofsf] c+ufnf]]sf kmfObfx? s]–s] x'g\ <  **-ax'pQ/_** | :tgkfg u/fpg ;lhnf] x'G5 | |  |
| --- | --- | --- | --- |
|  | aRrfn] k\|fs[lts Gofgf]kg kfpF5 | |  |
|  | aRrfsf] ;+s\|d0f 36fpF5 | |  |
|  | tf}n a9\g d2t u5{ |  |  |
|  | aRrfsf] cj:yf cg'udg ug{ ;lhnf] x'G5 | |  |
|  | cfdf / aRrfsf] ;DaGw k\|uf9 agfpF5 | |  |
|  | cGo -pNn]v ug]{_ ======================= | | |

| **^= >Ldfg / kl/jf/sf] :jLsfo{tf** | | | |
| --- | --- | --- | --- |
| ^=! s] tkfO{sf] >Ldfgn] klg dfofsf] c+ufnf]] lbg'x'G5 <  -tkfO{ vfgf vfFbf, 6\jfOn]6 hfFbf, g'xfpFbf, cf/fd ubf{ cflb_ | lbg'x'G5 | lbg'x'b}g | nfu" x'b}g |
| ^=@ s] tkfO{sf kl/jf/sf ;b:ox?n] klg dfofsf] c+ufnf]] lbg'x'G5 <  -tkfO{ vfgf vfFbf, 6\jfOn]6 hfFbf, g'xfpFbf, cf/fd ubf{ cflb_ | lbg'x'G5 | lbg'x'b}g | nfu" x'b}g |

| ^=# ;d'bfosf] dfofsf] c+ufnf]] k\|lt s] wf/0ff 5 < | ;xof]uL 7fg]sf] |  |
| --- | --- | --- |
|  | crDd dfg]sf] |  |
|  | a9L l;Sg vf]h]sf] |  |
|  | lvNnL p8fPsf] |  |
|  | yfxf 5}g |  |
|  | cGo ====================================== | |

| &= ;d:ofx? | | | | |
| --- | --- | --- | --- | --- |
| &=! dfofsf] c+ufnf]]sf] cEof; ubf{sf ;d:ofx? s] s] lyP < s[kof lj:tf/df atfOlbg'xf];\ .  ================================================================================================================================================================================================================================================= | | | | |
| *= ;Gt'i6L / l;kmfl/; | | | | |
| *=! tkfO{n] /f]Hg' ePsf] aRrfnfO{ Gofgf] kfg]{ sk8f k\|lt ;Gt'i6L x'g'x'G5 < | 5' *=# | | | 5}g |
| *=@ tkfO{ aRrfnfO{ Gofgf] kfg]{ sk8fk\|lt ;Gt'i6 gx'g'sf] sf/0f s] xf] < s[kof atfOlbg'xf];\.  ====================================================================================================================================================================================================================================================== | | | | |
| *=# s] tkfO{ dlxgf gk'uL hGd]sf÷sd tf}n hGd]sf aRrfx?sf] cfdfnfO{ dfofsf] c+ufnf]]sf] l;kmfl/; ug'{x'G5 < | | u5'{ | ulb{g | |
| sf/0f k\|f]j ug'{xf];\ | | | | |
| *=$ s] tkfO{ dlxgf gk'uL hGd]sf÷sd tf}n hGd]sf aRrfx?sf] cfdfnfO{ Gofgf] kfg]{ sk8fsf] l;kmfl/; ug'{x'G5 < | | u5'{ | ulb{g | |
| sf/0f k\|f]j ug'{xf];\  ============================================================================================================================================================================================================================================ | | | | |
| ;xefuLnfO{ cWoogsf] z'?b]lv lg/Gt/ ;xof]u u/]sf]df wGojfb lbg'xf];\ . pxfFn] dfofsf] c+ufnf]]sf] cEof; / aRrfnfO{ Gofgf] kfg]{ sk8fsf] af/]df dfly ul/Psf 5nkmndf 5'6]sf ljifox?df cGo dxTjk"0f{ wf/0ff, pkof]lutf / r'gf}tL jf s]xL s'/f÷egfO atfpg rfxg'x'G5 sL ;f]Wg'xf];\ . | | | | |

wGojfb
